# LMW cyclin E and its novel catalytic partner CDK5 are therapeutic targets and prognostic biomarkers in salivary gland cancers

**DOI:** 10.1038/s41389-021-00324-z

**Published:** 2021-05-14

**Authors:** Amriti R. Lulla, Said Akli, Cansu Karakas, Min Jin Ha, Natalie W. Fowlkes, Yoshitsugu Mitani, Tuyen Bui, Jing Wang, Xiayu Rao, Kelly K. Hunt, Laurent Meijer, Adel K. El-Naggar, Khandan Keyomarsi

**Affiliations:** 1 Departments of Experimental Radiation Oncology, The University of Texas MD Anderson Cancer Center, Houston, TX USA; 2 Departments of Biostatistics, The University of Texas MD Anderson Cancer Center, Houston, TX USA; 3 Departments of Veterinary Medicine and Surgery, The University of Texas MD Anderson Cancer Center, Houston, TX USA; 4 Departments of Pathology, The University of Texas MD Anderson Cancer Center, Houston, TX USA; 5 Departments of Bioinformatics and Computational Biology, The University of Texas MD Anderson Cancer Center, Houston, TX USA; 6 Departments of Breast Surgical Oncology, The University of Texas MD Anderson Cancer Center, Houston, TX USA; 7 ManRos Therapeutics & Perha Pharmaceuticals, Centre de Perharidy Roscoff, Roscoff, France

**Keywords:** Tumour biomarkers, Target identification, Oncogenes, Cancer models

## Abstract

Salivary gland cancers (SGCs) are rare yet aggressive malignancies with significant histological heterogeneity, which has made prediction of prognosis and development of targeted therapies challenging. In majority of patients, local recurrence and/or distant metastasis are common and systemic treatments have minimal impact on survival. Therefore, identification of novel targets for treatment that can also be used as predictors of recurrence for multiple histological subtypes of SGCs is an area of unmet need. In this study, we developed a novel transgenic mouse model of SGC, efficiently recapitulating the major histological subtype (adenocarcinomas of the parotid gland) of human SGC. CDK2 knock out (KO) mice crossed with MMTV-low molecular weight forms of cyclin E (LMW-E) mice generated the transgenic mouse models of SGC, which arise in the parotid region of the salivary gland, similar to the common site of origin seen in human SGCs. To identify the CDK2 independent catalytic partner(s) of LMW-E, we used LMW-E expressing cell lines in mass spectrometric analysis and subsequent biochemical validation in pull down assays. These studies revealed that in the absence of CDK2, LMW-E preferentially binds to CDK5. Molecular targeting of CDK5, using siRNA, resulted in inhibition of cell proliferation of human SGCs overexpressing LMW-E. We also provide clinical evidence of significant association of LMW-E/CDK5 co-expression and decreased recurrence free survival in human SGC. Immunohistochemical analysis of LMW-E and CDK5 in 424 patients representing each of the four major histological subtypes of human salivary cancers (Aci, AdCC, MEC, and SDC) revealed that LMW-E and CDK5 are concordantly (positive/positive or negative/negative) expressed in 70% of these patients. The co-expression of LMW-E/CDK5 (both positive) robustly predicts the likelihood of recurrence, regardless of the histological classification of these tumors. Collectively, our results suggest that CDK5 is a novel and targetable biomarker for the treatment of patients with SGC presenting with LMW-E overexpressing tumors.

## Introduction

Salivary gland cancers (SGCs) are rare malignancies with an incidence of 1/100,000 individuals in the United States. Age and gender play major roles in the incidence of SGCs, with the male-to-female incidence ratio being 1.6:1 and an increased incidence of ~7 cases/100,000 individuals aged ≥70 years^1^. Although SGCs are listed as part of head and neck cancers (HNCCs) (~1-6% of all HNCCs), the etiology of SGCs is not linked to smoking, alcohol consumption, environmental, and genetic factors as seen in HNCC tumors^2^.

Of the 30 subtypes, the most commonly encountered tumors in SGCs are mucoepidermoid (MEC, 45–70%)^3^, adenoid cystic (AdCC, 10–22%)^4^, acinic cell (Aci, 8–14%)^5^, and salivary duct carcinoma (SDC, 5–10%)^3^. Each of these subtypes significantly differ in their histological patterns, clinical behavior, and genetic alterations^3,5–7^, making SGCs a heterogeneous disease where standard staging parameters (tumor grade, TNM staging, etc.) have proved insufficient to predict prognosis and response to therapy. There is an unmet need for identifying biomarkers that can predict disease progression and also be therapeutically targeted, in more than one histologic subtype of salivary gland cancers.

Given the rarity of SGCs, animal models to study these tumors are critically needed, but not readily available. Some of the known mouse models reported include mice that develop tumors of subtypes SDC, Aci, and MEC. The SDC tumors in mice are inducible and driven by KRAS^G12D^ (*Ela-CreERT-LGL-KRAS*^*G12D*^), the most abundant mutation in this subtype^8^. A spontaneous Aci tumor model has been established with 100% penetrance using the MMTV-Cre/Apc^flox/flox^/Pten^flox/flox^ mice^9^. The most prevalent subtype, MEC, has been established in an *MMTV-RANKL* (NFkB ligand) and Justy (recessive mutation in the *Gon4l* gene) transgenic mouse models^10,11^. With the exception of SDC, these mouse models only recapitulate a fraction of the key drivers described in the human SGC tumors. Hence, there is a need to develop mouse models that can represent the most common SGC histological subtype that can be used for screening therapeutic vulnerabilities of these tumors.

In this study, we describe a novel mouse model of salivary gland cancer initiation (*MMTV-LMW-E; p53*+*/; CDK2−/−*) driven by the low-molecular-weight forms of cyclin E (LMW-E), the oncogenic properties of which have been reported by our group in both preclinical models and in patient samples, reviewed in ref. ^12^. These LMW-E murine adenocarcinomas of intermediate to high grade, arise primarily in the parotid region of the salivary gland, at par with majority of the salivary gland tumors observed clinically. Mechanistically, we have identified cyclin dependent kinase 5 (CDK5) as the primary and novel LMW-E associated CDK that can compensate for the absence of CDK2 to drive salivary gland tumorigenesis in these transgenic models. Using biochemical approaches, we demonstrate the binding of CDK5 to cyclin E in multiple in vitro and in vivo models. Further, using CDK5 siRNA, we show that salivary gland cancer cell lines are sensitive to inhibition of CDK5 as a function of LMW-E expression, but independent of CDK2 status. Lastly, evaluating a tissue microarray (TMA) from a cohort of 482 salivary gland cancer patients (424 evaluable for IHC) representing the Aci (*n* = 76), AdCC (*n* = 176), MEC (*n* = 80), and SDC (*n* = 92) subtypes, we show that the LMW-E and CDK5 concordant expression occurs in 70% of all patients and is strongly predictive of recurrence free survival.

## Results

### LMW-E drives salivary gland tumorigenesis, independent of CDK2 status

**Fig. 1:**
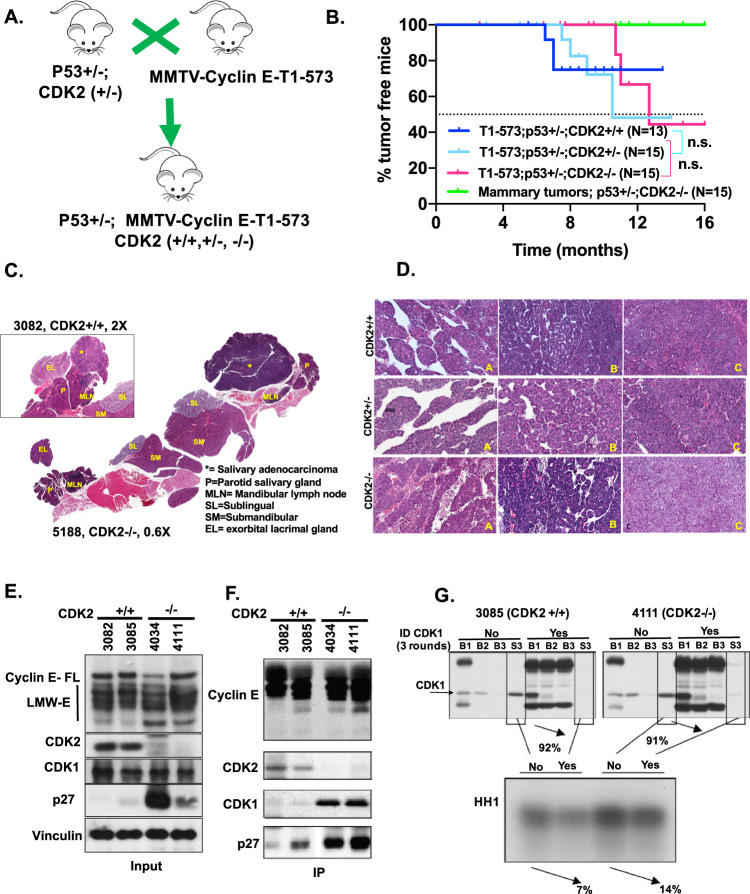
LMW-E drives salivary gland tumorigenesis, independent of CDK2 status. **A** Representative cross of transgenic MMTV-LMW-E-T1 and p53+/−, CDK2+/− to generate isogenic CDK2 (+/+, +/−, and −/−) MMTV-LMW-E-T1-p53+/− mice. **B** Percentage of tumor-free mice among MMTV-LMW-E-T1; p53+/−; isogenic for CDK2. CDK2+/+ (*n* = 13, dark blue line), CDK2+/− (*n* = 15, light blue line) and CDK2−/− (pink line, *n* = 12); compared to incidence of mammary tumors (green line, *n* = 12) in MMTV-LMW-E; p53+/−; CDK2−/− mice. Log-rank Mantel–Cox test was used to compare survival between the indicated genotypes. Samples sizes were estimated using the G power software. Data from our previous publication^17^ was used to estimate the differences between different CDK2 backgrounds. Mice were grouped by CDK2 genetic status without a need for randomization. **C** The murine parotid salivary gland (P) expanded and effaced by an adenocarcinoma (*). Unaffected salivary glands and adjacent structures including the sublingual salivary gland (SL), submandibular salivary gland (SM), exorbital lacrimal gland (EL), and mandibular lymph node (MLN) are shown in a CDK2−/− tumor (H&E, 0.6×). Inset: murine parotid salivary gland (P) expanded and effaced by an adenocarcinoma (*) in CDK2+/+ tumors (H&E, 2×). **D** Multiple histologic patterns within individual tumors and between tumors from CDK2+/+, CDK2+/−, and CDK2−/− mice. Typical histologic patterns including trabecular (**A**), acinar (**B**), and solid (**C**) (H&E, 20x). **E** Input (10%) using 25 μg protein was subject western blot analysis of salivary gland tumor lysates from MMTV-LMW; p53+/−; CDK2+/+ or CDK2−/− mice for the indicated markers. **F** Immunoprecipitation (IP) with cyclin E using 250 μg of salivary gland tumor lysates from (**E**) in MMTV-LMW; p53+/−; CDK2+/+ or CDK2−/− mice, followed by western blot analysis of the indicated markers. **G** Histone H1 (HH1) kinase assay to measure cyclin E associated kinase activity, with (Yes) or without (No) 3 rounds of immunodepletion (ID) of CDK1 from salivary gland tumor MMTV-LMW; p53+/−; CDK2+/+ (3085) or CDK2−/− (4111) mice. B1–B3 in both samples corresponds to beads after the first-third round of ID and S3 corresponds to the supernatant protein lysate used after the third round of ID showing >90% ID of CDK1.

Previously we reported that the oncogenic forms of cyclin E, LMW-E, are strong predictors of poor outcome and resistance to chemotherapy in breast cancer patients^13–15^. LMW-E binds more strongly to CDK2 compared to full-length cyclin E and is resistant to inhibition by CDK inhibitors (CKI)^16^. Further, LMW-E/CDK2 interaction is required to drive mammary gland tumorigenesis in transgenic mouse models^17^. Here, we report that LMW-E can also drive the initiation of salivary gland tumors (SGTs), but independently of CDK2 status (Fig. 1A, B). Specifically, SGTs are prevalent with a comparable frequency of 25% (3/12) in the MMTV-LMW-E mice when CDK2 is genetically knocked out (i.e., CDK2−/−) as compared to 23.1% (3/13) in CDK2+/+ and 26.7% (5/15) in CDK2+/− backgrounds, respectively (Fig. 1B). Histologically, the tumors in all three genetic backgrounds (i.e., CDK2+/+, CDK2+/−, and CDK2−/−) are predominantly adenocarcinomas, which are commonly found in the parotid gland^18^. Most of these murine adenocarcinomas are intermediate to high grade tumors. Other salivary glands including the sublingual salivary (SL), submandibular salivary (SM), and minor glands were unaffected (Fig. 1C). The three main histological patterns within each tumor included ductal (Fig. 1D, inset A), acinar (Fig. 1D, inset B) and solid forms (Fig. 1D, inset C). SGTs also showed comparable number of mitotic figures, regardless of CDK2 status (Supplementary Fig. 1A). Collectively, these results suggest that the development of salivary gland adenocarcinomas initiated by LMW-E in our transgenic mouse models is independent of CDK2 status.

### CDK1 partially rescues the LMW-E associated kinase activity in the absence of CDK2

We next examined if the functionally redundant CDK1^19^ could substitute for the loss of CDK2 and bind to LMW-E to drive salivary gland tumorigenesis. To test this hypothesis, we compared SGTs from CDK2+/+ and CDK2−/− genetic backgrounds and found that the absence of CDK2 does not affect cyclin E expression (Fig. 1E). However, expression of p27, a CKI, was higher in the SGTs from the CDK2−/− compared to CDK2+/+ backgrounds, suggesting that this CKI may be interacting with another LMW-E bound CDK (Fig. 1E). Examination of cyclin E immune complexes showed that LMW-E preferentially binds to both CDK1 and p27 in CDK2−/− as compared to CDK2+/+ tumors (Fig. 1F). To assess if CDK1 can rescue the cyclin E associated kinase activity in CDK2−/− tumors, we immunodepleted CDK1 using three rounds of immunodepletion, which was sufficient to reduce CDK1 levels by 90% (Fig. 1G, upper panel, compare S3 lanes). Cyclin E-associated kinase activity in these CDK1 immunodepleted SGT lysates decreased only by 7% and 14% in the CDK2+/+ (Fig. 1G, sample 3085) and CDK2−/− (Fig. 1G, sample 4111) backgrounds respectively. This suggests that CDK1 is not the main kinase interactor for LMW-E in these salivary gland-derived-transgenic tumors. Pharmacological inhibition of CDKs by 5 µM roscovitine (a pan-CDK inhibitor selective for CDKs 1, 2, 7, and 9^20^), also proved insufficient to rescue the cyclin-E associated kinase activity in the CDK2−/− tumors as compared to CDK2+/+ tumors (Supplementary Fig. 1B). Only a high and off-target dose of roscovitine (80 μM) was able to reduce the cyclin-E associated kinase activity to 60% in CDK2−/− tumors (sample 4111, Supplementary Fig. 1C). Collectively, this genetic ablation and pharmacological studies suggest that in the absence of CDK2, LMW-E is likely to associate with other CDKs, beyond CDK1.

### CDK5 binds to LMW-E, independent of CDK2 status

**Fig. 2:**
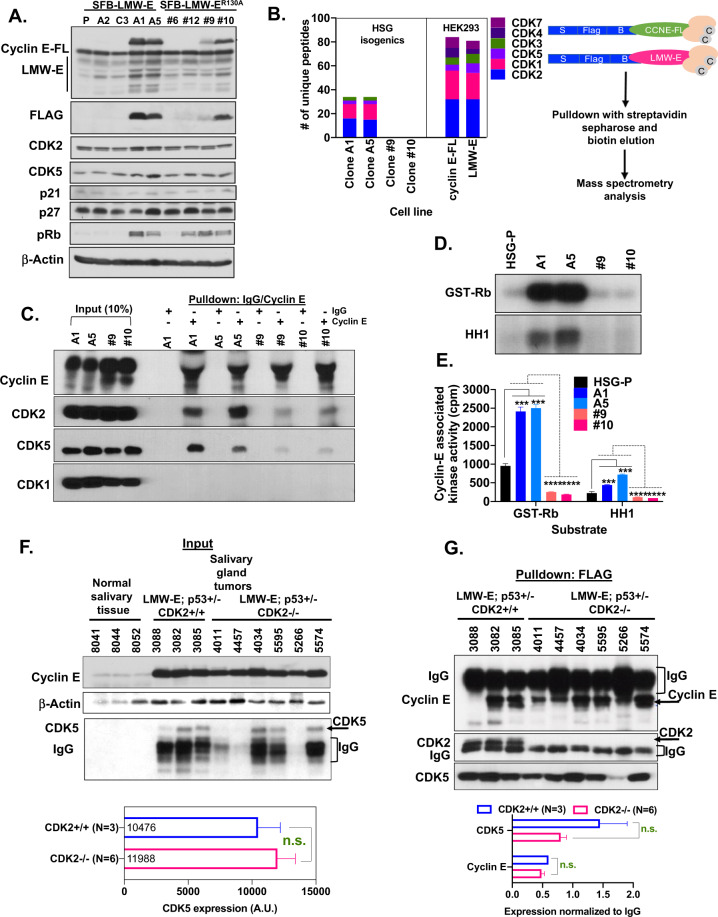
CDK5 binds to LMW-E in salivary gland cell lines and transgenic tumor tissues independently of CDK2. **A** Western blot analysis showing LMW-E expression and other cell cycle markers in isogenic human salivary gland (HSG) clones. Lentivirus transduction was used to generate cells stably expressing either S protein-FLAG-Streptavidin binding protein (SFB) tagged LMW-E or CDK2 binding site mutated LMW-E^R130A^. **B** (Left panel) Isogenic HSG cell lines and HEK293 were subjected to immunoprecipitation (IP) with an anti-flag antibody using 1 mg of protein extracts followed by flag peptide elution and loading (20% of eluent) onto a 10% SDS-PAGE followed by mass spectrometry analysis. The bar graphs show the number of unique peptides of CDKs each protein bound to either LMW-E expressing clones A1 and A5 or LMW-E^R130A^ expressing clones 9 and 10 and cyclin E full length and LMW-E overexpressing HEK-203 cells. (Right panel) Schematic of the SFB constructs used to generate stable isogenic HEK293 and HSG cell lines expressing either full-length cyclin E (cyclin E-FL), LWM-E or CDK2 binding site mutated LMW-E^R130A^ constructs. **C** IP/western blot analysis in LMW-E expressing clones A1 and A5 and LMW-E^R130A^ expressing clones #9 and #10. Briefly, 400 µg protein lysates from each cell line were subjected to IP with either anti-IgG or anti-cyclin E and the indicated cyclin E bound proteins were assessed by western blot analysis. Input lanes were loaded with 40 µg of whole-cell lysates for each cell line. Pulldown lanes labeled IgG lanes indicate pulldown with anti-IgG and lanes labeled cyclin E indicate pulldown with anti-cyclin E. **D** Radiographic film showing the phosphorylation of the substrates GST-Rb and HH1 following IP with cyclin E, using 400 µg protein extracts of the indicated cell lines. **E** Quantitation of the bands from (D). The bar graph is the quantification of the mean cyclin E associated kinase activity, measured as counts per million (cpm) radioactivity units, representative of three independent kinase assay experiments. *p* Values (**p < 0.01, ***p < 0.001, ********p < 0.0001) were calculated using the unpaired t-test with Welch’s correction. **F** Western blots analysis indicating unchanged expression of CDK5 in tumors from derived from LMW; p53+/−; CDK2+/+ mice (*N* = 3) and LMW; p53+/−; CDK2−/− mice (*N* = 6). The bottom panel depicts the ImageJ quantification (and comparison) of CDK5 between the CDK2+/+ (*N* = 3) and CDK2−/− (*N* = 6) tumors. **G** Immunoprecipitation with anti-FLAG followed by western blot analysis to assess the binding of CDK5 and CDK2 to LMW-E in salivary gland tumors from LMW-E; p53+/−; CDK2−/− (*N* = 3) and LMW-E; p53+/−; CDK2+/+ (*N* = 6) mice. The bottom panel depicts the ImageJ quantification (and comparison) of cyclin E and CDK5 IP/western bands between the CDK2+/+ (*N* = 3) and CDK2−/− (*N* = 6) tumors.

To identify LMW-E associated binding proteins, isogenic human salivary gland (HSG) cell lines stably expressing either the LMW-E or CDK2 binding site mutated LMW-E^R130A^ FLAG-tagged-proteins were generated (Fig. 2A). The overexpression of LMW-E (clones A1 and A5) and LMW-E^R130A^ (clones #9 and #10) did not alter the expression of other cell cycle-regulated proteins examined (Fig. 2A). The HSG clones (i.e., A1, A5, clone 9 and clone 10) and HEK293 cells [transiently overexpressing either full-length cyclin E (cyclin E-FL) or LMW-E] were subjected to immunoprecipitation with cyclin E (anti-FLAG) followed by mass spectrometry analysis using the S protein-FLAG-Streptavidin binding protein (SFB) elution system (Fig. 2B, schema). CDK1 and CDK2 were the predominant CDK peptides bound to both cyclin E-FL (*N* = 24 for CDK1 and *N* = 32 peptides for CDK2) and LMW-E containing lines A1, A5, and HEK293 (*N* = 12,13, and 22 for CDK1 and *N* = 16, 15, and 32 peptides for CDK2, respectively) (Fig. 2B, Supplementary Fig. 2A). Mutation in the CDK binding domain of cyclin E at R130A abolished binding of all CDKs and limited the number of other cell-cycle associated proteins bound to LMW-E (Fig. 2B, Supplementary Fig. 2A). Beyond CDK1 and CDK2, mass spectrometry results revealed CDK5 to also bind to cyclin E in both the HEK293 and isogenic HSG systems (*N* = 3 peptides in all HSG cell lines and *N* = 5 and 8 peptides for cyclin E-FL and LMW-E, respectively in HEK293 cells) (Fig. 2B and Supplementary Fig. 2A, B). To confirm the newly identified LMW-E-CDK5 interaction, we performed immunoprecipitation (IP) followed by western blot analysis with CDK5 using isogenic LMW-E cell lines. Cyclin E IP/western blot analysis revealed that the LMW-E forms in the A1 and A5 clones can bind to CDK5, (see LMW-E lines A1 and A5 in Fig. 2C), while the binding of CDK5 to LMW-E in the LMW-E^R130A^ mutants #9 and #10 was significantly reduced (see LMW-E lines #9 and #10 in Fig. 2C), consistent with the mass spectrometry data. Lastly, to verify the specificity of the cyclin E-CDK5 interaction, we probed for unbound proteins in the supernatants of the cyclin E IP’ed samples that were immunodepleted for cyclin E (following 3 rounds of anti-cyclin E IP). CDK1, CDK4, and CDK6 were left unbound (see IP Cyclin E lanes in Supplementary Fig. 2D), indicating that these CDKs do not bind to LMW-E in the salivary gland cell lines examined. Given the predominant cyclin E-CDK5 interaction in the HSG cell lines, we next asked if mutating the CDK binding site on cyclin E had any impact on the cyclin E associated kinase activity in these cell lines. As seen in Fig. 2D, E, both the GST-Rb and HH1 kinase substrates, showed maximum phosphorylation when cyclin E was present in its low molecular weight forms (i.e., A1 and A5). No cyclin E-dependent kinase activity was observed in mutant lines #9 and #10.

Next, to interrogate if the cyclin E-CDK5 interaction occurs independently of CDK2, we examined the nature of expression and binding of cyclin E to CDK5 in SGT from our transgenic models. Results revealed no significant changes in the expression of cyclin E and CDK5 between CDK2+/+ (*n* = 3) and CDK2−/− (*n* = 6) compared to normal salivary gland tissues (*N* = 3) (Fig. 2F). IP/western analysis of cyclin E/CDK5 in the same tumors from Fig. 2F further confirmed that CDK5 binds to cyclin E, independently of CDK2 status (Fig. 2G, see bottom panel for quantification).

**Fig. 3:**
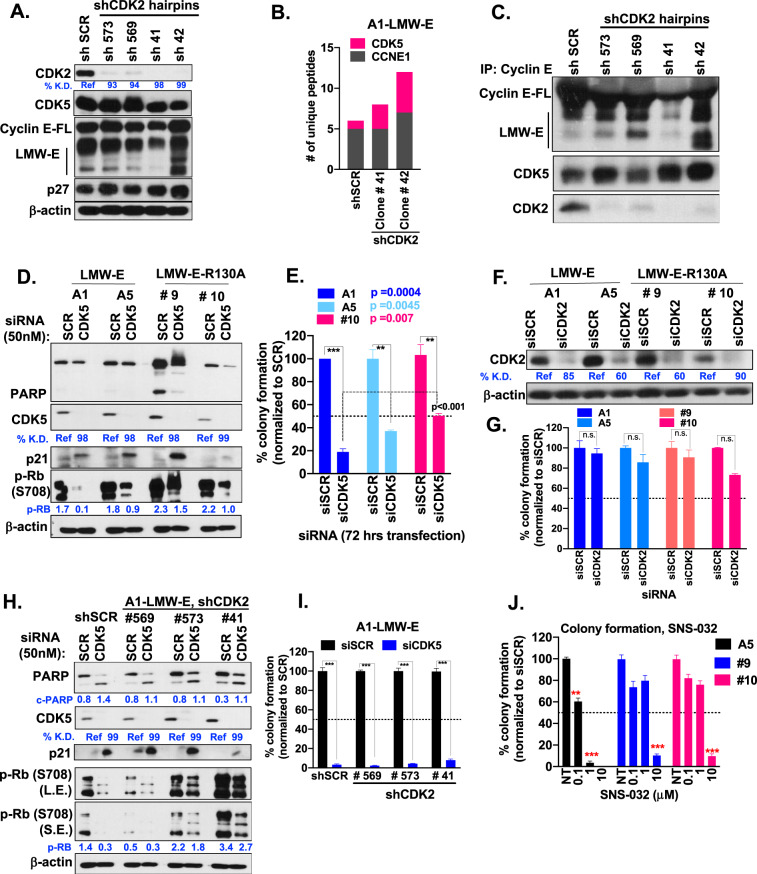
CDK5 is a putative druggable target in human salivary gland cell lines. **A** Western blot analysis showing the efficiency of CDK2 knockdown (KD) with four different shRNA hairpins in LWM-E expressing A1 cell lines. Numbers indicate the % KD, quantitated using ImageJ and normalized to the loading control β-actin. **B** Mass spectrometry results indicating the number of unique peptides of CDK5 bound to LMW-E in A1 cell lines, stably transfected with either shScramble (shSCR) or two different hairpins of shCDK2. **C** IP/western blots analysis assessing the binding of CDK2 and CDK5 in A1-LMW-E cell lines (from Panel **A**), with and without stable knockdown of CDK2. Briefly, 400 μg of protein from each cell line were subjected to IP with anti-cyclin E (1 μg antibody per 500 μg protein) overnight, followed by western blot analysis of the indicated proteins. **D**, **H** Isogenic HSG cell lines (A1, A5, clones 9 and 10) and A1 isogenic cell lines (−/+ shCDK2 knockdown) were transfected with either 50 nM of SCR siRNA or CDK5 siRNA. Seventy-two-hours post transfection, cells were collected, counted, and assessed for cell death markers by western blot analysis. Numbers indicate the percent KD (for CDK5) and expression (PARP and p-Rb) quantitated using ImageJ and normalized to the loading control β-actin. **E**, **I** Five hundred cells per well from cells transfected in (**D**) and (**H**) were replated for colony formation assay in six-well plates (triplicates/sample). Nine-days post plating, colony formation was assessed by crystal violet staining and colonies enumerated. Percent colony formation represents the mean of 3 independent experiments and was calculated by normalizing colony counts to the SCR wells/cell line. *p* Values (*p < 0.05, **p < 0.01, ***p < 0.001) were calculated using the unpaired *t* test with Welch’s correction. **F** Isogenic HSG cell lines (LMW-E expressing A1 and A5, LMW-E^R130A^ expressing clones 9 and 10) were transfected with either 50 nM of SCR siRNA or CDK2 siRNA. Seventy-two hours post transfection, cells were collected, counted and assessed for KD by western blot analysis. Numbers indicate % KD of CDK2, normalized to β-actin. **G** Five hundred cells/well from cells transfected in (**F**) were replated for colony formation assay in six-well plates (triplicates/sample). Nine-days post plating, colony formation was assessed by crystal violet staining, and colonies were enumerated. Percent colony formation represents the mean of two independent experiments and was calculated by normalizing colony counts to the SCR wells/cell line. **J** Isogenic HSG cell lines (LMW-E expressing A1 and A5, LMW-E-R130A expressing clones 9 and 10) were treated with 0.1, 1, and 10 μM (triplicates/sample/dose) of SNS-032 for 3 days. Thereafter, cells were collected and plated in 6- (colony forming assays) well plates at a density of 500 cells/well for 12 days with the drug. Media was replenished every 3 days. Effect on cell proliferation was assessed using crystal violet staining, colonies were enumerated and presented as bar graphs respectively. % colony formation represents the mean of three independent experiments. *p* Values (*p < 0.05, **p < 0.01, ***p < 0.001) were calculated using the unpaired *t* test with Welch’s correction.

The above findings in SGTs were further validated using LMW-E cell line A1, stably knocked down (KD) for CDK2 using four different shRNA hairpins (Fig. 3A). FLAG IP/mass spectrometry analysis in these cells showed that in the absence of CDK2, CDK5 is the major kinase that binds to LMW-E (Fig. 3B). Cyclin E IP/western blot analysis in the shCDK2 cells further confirmed the mass spectrometry results revealing that the binding of CDK5 to LMW-E occurred regardless of CDK2 status (Fig. 3C). Taken together, these results have identified a novel cyclin E-CDK5 interaction that occurs independently of CDK2 status in SGT cell lines and tumors derived from our transgenic models.

### LMW-E overexpressing salivary gland tumor cell lines are sensitive to CDK5 inhibition, independent of CDK2 status

Next, to interrogate if the disruption of the LMW-E-CDK5 interaction has functional effects on HSG cell lines, we knocked down CDK5 and examined its consequences on cell cycle and colony-forming assays. Transient knockdown of CDK5 (via siRNA) resulted in a 10% increase in the G0/G1 phase of the cell cycle (Supplementary Fig. 3A, B), induction of p21, and reduced levels of p-Rb (Fig. 3C). No significant cell death was observed as assessed by lack of PARP cleavage in western blot analysis (Fig. 3C) or caspase 3 (data not shown). Thereafter, to assess effects on viability, cells transfected with CDK5 siRNA for 3 days, washed thereafter and re-plated for colony formation assay. Crystal violet stained plates in Supplementary Fig. 3C, showed a significant reduction in the number of colonies in the CDK5 siRNA conditions with a 70% reduction in colony-forming ability of the LMW-E overexpressing cell lines (A1 and A5) compared to 43% in the LMW- E^R130A^ #10 cell line (Fig. 3E). Next, we examined if knockdown of CDK2 augmented the functional effects seen with CDK5 inhibition. Knockdown of CDK2 alone had no impact on the colony-forming ability of the LMW-E cell lines (A1 and A5) or LMW- E^R130A^ mutant lines #9 and #10 (Fig. 3F, G). Next, we used the shCDK2 cell lines (see Fig. 3A for CDK2 knockdown) to transiently downregulate CDK5 using siRNA. Induction of p21 and reduced levels of p-Rb were observed, along with a consistent G1-arrest, independent of CDK2 status (Fig. 3H, Supplementary Fig. 3D, E). Furthermore, colony-forming assays showed no differences between the CDK5 siRNA groups of shSCR compared to shCDK2 lines, i.e., regardless of CDK2 status, all cell lines examined had equal sensitivity to CDK5 inhibition (Fig. 3I and Supplementary Fig. 4A). Treatment of HSG cell lines with a CDK2 inhibitor SNS-032 resulted in EC_50_ of 0.15 μM in all LMW-E cell lines (A1 and A5) (Supplementary Fig. 4B). However, given the selectivity of SNS-032 to additional CDKs (CDK7 EC_50_ = 0.62 μM and CDK7 EC_50_ = 0.04 μM)^21,22^, we validated the dose–response results with colony formation assays (Fig. 3J). Totally, 0.1 μM SNS-032 (EC_50_ dose) reduced the colony formation ability of LMW-E expressing A5, LMW-E^R130A^ mutant lines #9 and #10 by 40% and 25% each, respectively. A dose of 1 μM SNS-032 (10-fold above EC_50_) further reduced the colony counts to 90% in A5-LMW-E, compared to 25–30% seen in LMW-E^R130A^ mutant lines #9 and #10, validating the LMW-E associated kinase data in Fig. 2D, E. Thus, pharmacological inhibition with CDK2 has modest functional effects on the salivary gland cell lines at low doses and demonstrated off-target growth inhibition at higher doses. To further assess if the effects of CDK5 inhibition are specific and not a consequence of the knockdown of a kinase; we examined the role of other CDK inhibitors. LMW-E and LMW-E^R130A^ mutant lines treated with the CDK4/6 inhibitor palbociclib showed no response (Supplementary Fig. 4C), consistent with our previously published work^23^, where we have shown that LMW-E is a marker of palbociclib resistance. Similarly, the lack of on-target response to CDK1 inhibitor Ro-3306 (Supplementary Fig. 4D, E) is consistent with our cyclin E IP/western blot analysis findings in Fig. 2D. Taken altogether, our results show that CDK5 is the major LMW-E associated kinase, required for the proliferation of SGT cell lines.

### LMW-E and CDK5 are concurrently overexpressed in HSG tumors

**Fig. 4:**
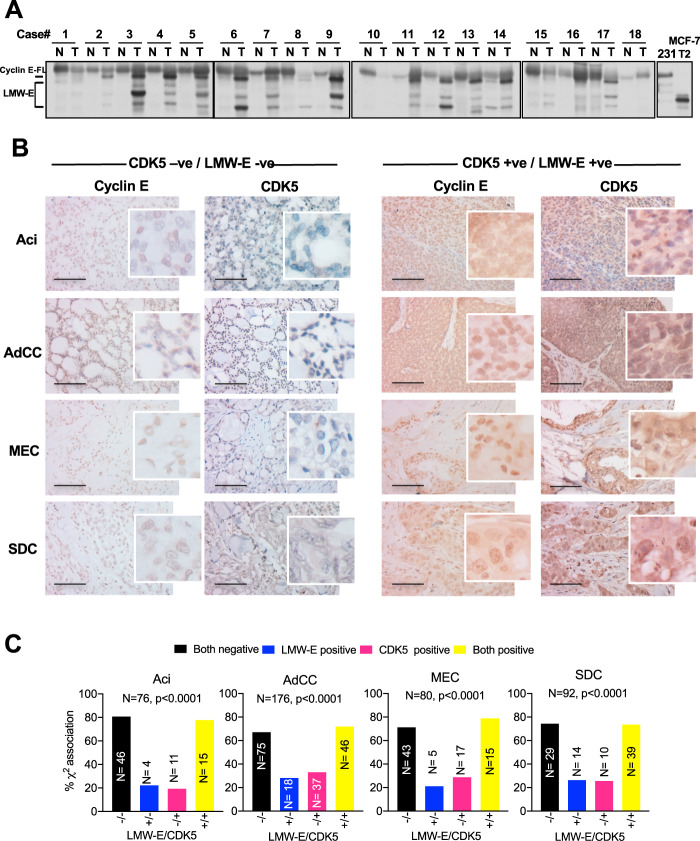
LMW-E and CDK5 are co-expressed in human salivary gland tumors. **A** Western blot analysis of cyclin E, in 18 salivary gland tumor samples from patients of different histological subtypes. Lane designations correspond to sample numbers given in Supplementary Table 1.231: MDA-MB-231 parental cells; MCF-T2: MCF-7 cells transfected with LMW-E (T2 isoform) **B** Analysis of cyclin E and CDK5 expression in different histologic subtypes of salivary duct carcinomas. Representative immunohistochemistry images for CDK5 and cyclin E are shown. Original magnification: ×200 (main images); ×800 (insets). Specifically, cyclin E (A1–D1) and corresponding CDK5 (A2–D2) staining for low cyclin E/CDK5; cyclin E (A3–D3) and corresponding CDK5 (A4–D4), for high CDK5/cyclin E expression. **C** Correlation analysis between high/low cyclin E (cytoplasmic staining) and CDK5 expression in salivary gland tumors samples of patients from four different subtypes. Black bar = both negative/ low expression of LMW-E and CDK5, blue bar = one positive/high LMW-E expression only, pink bar = one positive/high CDK5 expression only and yellow bar = both positive/high expression of both LMW-E and CDK5. *p* Values indicate a significant correlation between cyclin E (cytoplasmic staining) and CDK5 expression and were calculated by the Chi-square ($\chi^2$) test of association.

To translate our findings from mouse transgenic models to human samples, we examined the expression of LMW-E initially by western blot analysis in 18 human primary tumors corresponding to different histological subtypes of SGTs with Aci (7/18) being the major histological subtype observed in this cohort (Supplementary Table 1). Western blot analysis indicated that 61% (11/18) SGTs expressed LMW-E exclusively in the tumor tissue compared to normal adjacent tissue samples and that such expression was not confined to a specific histological subtype (Fig. 4A, Supplementary Table 1).

Next, we examined cyclin E and CDK5 by IHC in a cohort of 482 patients using a TMA, representing the four subtypes of Aci (*N* = 80), AdCC (*N* = 215), MEC (*N* = 82), and SDC (*N* = 105), respectively. A flow chart depicting the composition of the study group for each subtype is depicted in Supplementary Fig. 5. Table 1 summarizes the clinical and pathological characteristics for each subtype of the study population. The majority of the patients in all four subtypes presented with a high tumor grade, with low incidence of perineural and lymphovascular invasion. When assessing primary tumor sites, both Aci and SDC tumors were prevalent in the parotid gland with frequencies of 92.5% and 78.1%, respectively (Table 1). AdCC tumors presented with comparable frequencies of 25.1% and 26.1% in the major salivary glands and the maxilla, respectively (Table 1 and Supplementary Table 2), MEC tumors were predominant in the major salivary glands (37.8% frequency), followed by tongue and maxilla [17.1% and 12.2% frequencies, respectively (Supplementary Table 2)].

**Table 1:** Summary of clinicopathologic variables in salivary gland cancer patients.

Variable	Aci (*N* = 80)		AdCC (*N* = 215)		MEC (*N* = 82)		SDC (*N* = 105)	
	No. (%)	*p* Value	No. (%)	*p* Value	No. (%)	*p* Value	No. (%)	*p* Value
*Age, years*								
Median	60	0.1123	62	0.9492	63.5	0.3073	65	0.01517
Mean (range)	60 (23–90)		63 (27–96)		61 (22–97)		66 (37–96)	
*Sex*								
Female	47 (60.26)	0.1152	113 (52.56)	0.732	51 (62.2)	0.0439	36 (34.29)	0.0001
Male	31 (39.74)		102 (47.44)		31 (37.8)		69 (65.71)	
*Race*								
White	63 (78.75)	0.8144*	160 (74.42)	0.08771	60 (73.2)	0.2126	95 (90.48)	0.0029
Black	3 (3.75)		16 (7.44)		3 (3.7)		4 (3.81)	
Hispanic	11 (13.75)		29 (13.49)		15 (18.3)		5 (4.76)	
Others	3 (3.75)		10 (4.65)		4 (4.9)		2 (0.95)	
*Tumor size (CM)*								
Median	2.3	0.03424	3	0.4788	2	0.05264	3	0.01175
Mean (range)	2.6 (0.8–6.5)		3.1 (0.5–15)		2.7 (1–6)		3.4 (0.7–10)	
*Tumor grade*								
Low	4 (15.4)	0.0142**	13 (14.13)	<0.0001	15 (18.7)	0.0003	3 (4.11)	<0.0001
Intermediate	2 (7.7)		50 (54.35)		40 (50.0)		8 (10.96)	
High	20 (76.9)		29 (31.52)		25 (31.3)		62 (84.93)	
*Perineural invasion*								
No	54 (77.14)	<0.0001	56 (26.42)	<0.0001	65 (80.3)	<0.0001	39 (41.94)	0.334
Yes	16 (22.86)		156 (73.58)		16 (19.7)		54 (58.06)	
*Lymphovascular invasion*								
No	62 (88.57)	0.07545	178 (84.36)	0.0431	68 (83.9)	0.414	57 (60.64)	<0.0001
Yes	8 (11.43)		33 (15.64)		13 (16.1)		37 (39.36)	
*Margin*								
Negative	51 (75.0)	0.05616	102 (49.28)	<0.0001	61 (74.4)	0.04181	76 (79.17)	0.0007
Positive	17 (25.0)		105 (50.72)		21 (25.6)		20 (20.83)	
*LMW-E (H-score)*								
0	57 (75.0)	0.01799	113 (63.13)	0.8748	60 (75.0)	0.01437	39 (40.62)	<0.0001
1	19 (25.0)		66 (36.87)		20 (25.0)		57 (59.38)	
*CDK5 H-score*								
0	50 (65.79)	0.03849	95 (52.49)	0.5621	47 (59.5)	0.3783	43 (44.79)	0.0427
1	26 (34.21)		86 (47.51)		32 (40.5)		53 (55.21)	
*Clinical TNM stage*								
I	15 (23.08)	0.2042	13 (6.7)	0.0003	22 (28.6)	<0.0001	12 (13.33)	0.0021
II	10 (15.38)		33 (17.01)		21 (27.3)		4 (4.44)	
III	7 (10.77)		27 (13.92)		6 (7.8)		8 (8.89)	
IV	33 (50.77)		121 (62.37)		28 (36.3)		66 (73.33)	
*Combined LMW-E and CDK5 (H-score)*								
Both negative	46 (75.41)	0.02247	75 (61.98)	1	43 (53.8)	0.04726	29 (40.28)	<0.0001
Both positive	15 (24.59)		46 (38.02)		15 (18.7)		43 (59.72)	
*Primary tumor site*^a^								
Parotid gland	74 (92.5)	<0.0001	31 (14.4)	<0.0001	27 (32.9)	0.0098	82 (78.1)	<0.0001
Submandibular gland	2 (2.5)		21 (9.8)		3 (3.7)		8 (7.6)	
Sublingual gland	0 (0)		2 (0.9)		1 (1.2)		0 (0)	
Maxilla and maxillary sinuses	0 (0)		56 (26.1)		10 (12.2)		4 (3.8)	
Others	4 (5.0)		105 (48.8)		41 (50.0)		11 (10.5)	

*p Value after combining “Black” and “Others” categories.

**p Value after combining “Low” and “Intermediate” categories.

^a^Supplementary Table 2 lists all the sites.

Summary of clinicopathologic variables in patients with Aci (*N* = 80), AdCC (*N* = 215), MEC (*N* = 82), and SDC (*N* = 105) subtypes of salivary gland primary tumors from patients. For tests of homogeneity of each subtype, one-way ANOVA and chi-squared tests were used to compare distributions of continuous and categorical factors between the subtype group and other subtypes (pooled).

IHC staining and scoring of cyclin E and CDK5 (on 424 evaluable samples) stratified patients as low/high (*H* score 0/1, respectively). In the case of LMW-E, a score of 1 indicated the presence of cyclin E primarily in the cytoplasm, which represents LMW-E lacking a nuclear localization signal as we previously reported^14,15,24–27^. Figure 4B represents images of the immunostaining patterns of low (Fig. 4B, left) and high (Fig. 4B, right) LMW-E and CDK5 expression in each of the four SGT subtypes. For each subtype, the single expression of LMW-E or CDK5 was detected in only 19-31% of all the patients (Fig. 4C, blue and pink bar graphs). However, when we assessed the co-expression (or lack of co-expression) of LMW-E and CDK5, 68–80% of all patients showed a significant concordance in the expression of these biomarkers (Fig. 4C, yellow and black bar graphs).

### LMW-E and CDK5 are associated with poor recurrence-free survival (RFS), independent of histological subtypes of salivary gland

**Fig. 5:**
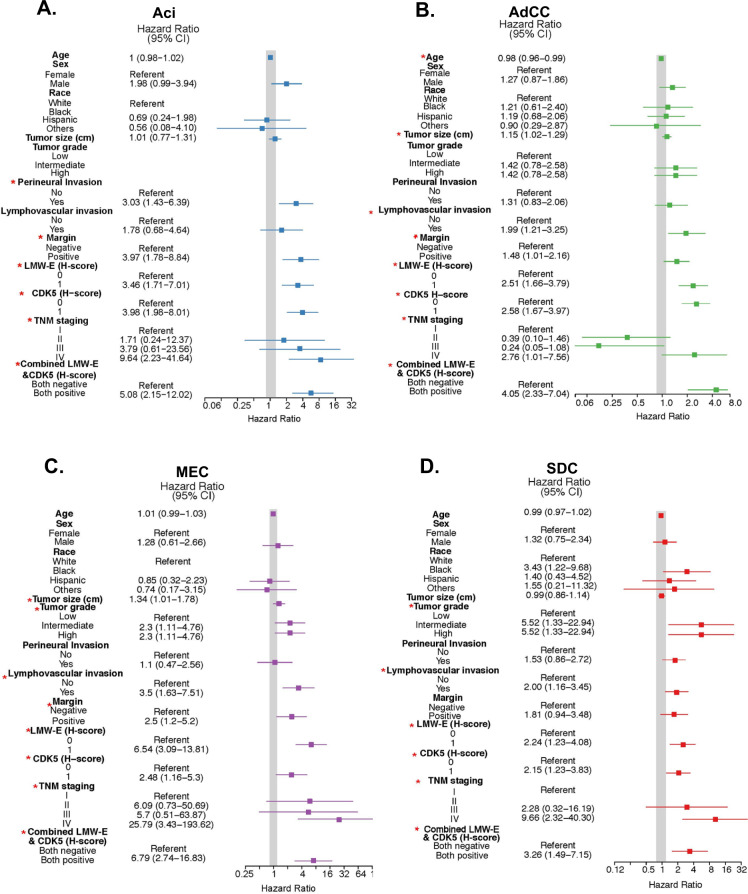
LMW-E and CDK5 are associated with recurrence-free survival in all subtypes of salivary gland tumors. Forest plots analysis indicating univariable analysis of the indicated variables with recurrence-free survival in each subtype. The solid gray line indicates a hazard ratio of 1 for the overall population. Variables with *p* values <0.05 are marked with * and are significant predictors of recurrence-free survival for each subtype, calculated using the Cox proportional-hazards model.

**Fig. 6:**
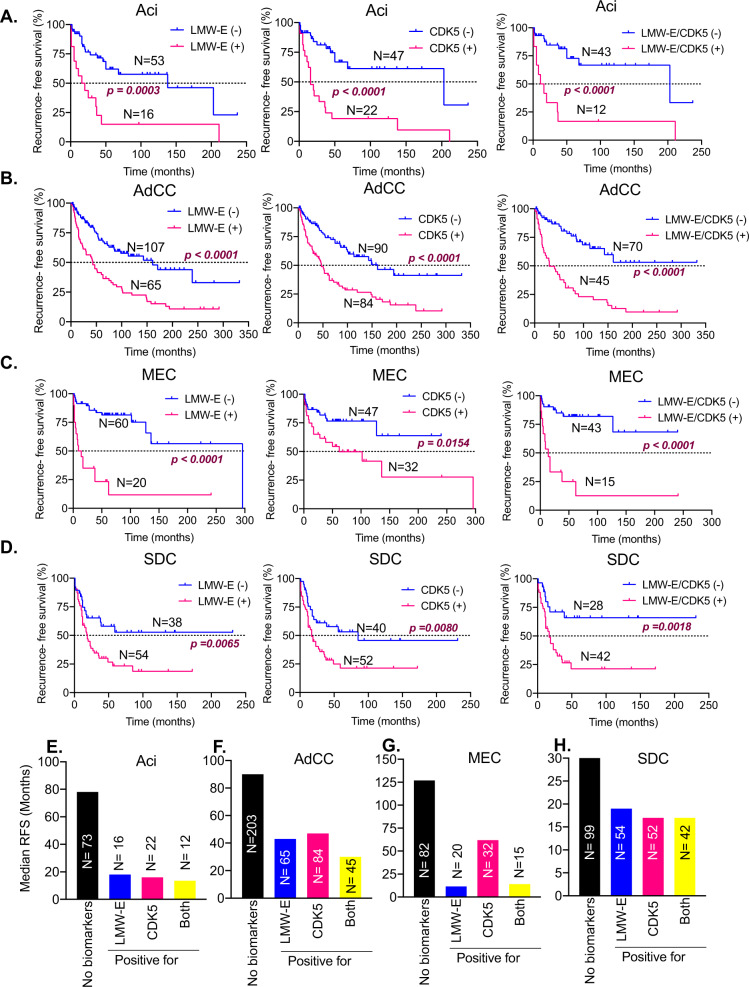
High Cyclin E and CDK5 expression can predict recurrence in all subtypes of salivary gland tumors. **A**–**D** Kaplan–Meier plots for subtypes Aci, AdCC, SDC, and MEC, respectively, according to LMW-E, CDK5, or LMW-E/CDK5 expression. The expression of LMW-E and CDK5, individually and combined, were significantly associated with decreased time to recurrence in all the cohorts. *p* Values indicate comparison between survival curves, calculated using the log-rank (Mantel–Cox) test. **E**–**H**. Graph comparing median RFS without any biomarkers (black bars) to that when patient tumor samples are positive (*H*-score = 1) for LMW-E (blue bars), CDK5 (pink bars), or LMW-E /CDK5 (yellow bars) for each subtype.

To determine if high expression of LMW-E and CDK5 are associated with RFS, we performed the univariable analysis with LMW-E and CDK5 as well as standard markers of clinical outcome such as tumor grade and TNM staging (Fig. 5A–D and Supplementary Table 3). Of the 482 patients identified, 67 were excluded due to insufficient or missing tissue for IHC or lack of availability of outcome data due to loss of follow-up (see flow chart in Supplementary Fig. 5). The IHC staining of LMW-E and CDK5 in the remaining 415 patients (Aci, *N* = 69; AdCC = 174; MEC, *N* = 80 and SDC, *N* = 92) (Supplementary Fig. 5) reveals that high expression of LMW-E or CDK5 (*H*-score = 1) was significantly associated with a high probability of recurrence, independent of subtype. The hazard ratios (HRs) ranged from 2.24 to 6.54 and 2.15 to 3.98 across subtypes for LMW-E and CDK5, respectively. These HRs were higher than that of high tumor grade (HR = 1.42–5.52), lymphovascular invasion (HR = 1.78–3.5), and positive margins (HR = 1.48–3.97). With a concurrent high expression of LMW-E and CDK5 (both positive), the HRs further increased to 3.26–6.79 across subtypes and were significant predictors of poor RFS (Fig. 5A–D and Supplementary Table 3). Among the different subtypes, MEC patients presented with the highest risk of recurrence with concurrent LMW-E and CDK5 high expression (HR = 6.79) (Fig. 5C). Given this strong association of LMW-E and CDK5 expression with poor RFS, we examined the time to recurrence as a function of individual and combined LMW-E and CDK5 expression. Median RFS follow-up time for the four cohorts, without taking into account the impact of either LMW-E or CDK5, were 6.5 (Aci), 7.5 (ADCC), 10.58 (MEC), and 2.75 (SDC) years (Supplementary Table 4). When the expression of LMW-E and CDK5 were factored in, high individual and combined expression of LMW-E and CDK5 significantly reduced median RFS in all subtypes (Fig. 6A–D). Specifically, patients with high LMW-E expression showed a 3.7–25.7-fold reduced median RFS compared to low LMW-E patients (Supplementary Table 4). In patients with high CDK5 expression, a similar 3.4–12.7-fold reduced median RFS was observed. When co-overexpressed, the LMW-E/CDK5 (+) patients showed an accelerated time to recurrence by 6–15-fold (i.e., 13.5–30 months) compared to LMW-E/CDK5(−) patients (>203 months) (Supplementary Table 4). Thus, LMW-E and CDK5 are robust biomarkers to predict RFS and their high co-overexpression (yellow bars) are associated with an accelerated time to recurrence by 2.3–6.1-fold as compared to no biomarkers (black bars) (Fig. 6E–H).

**Fig. 7:**
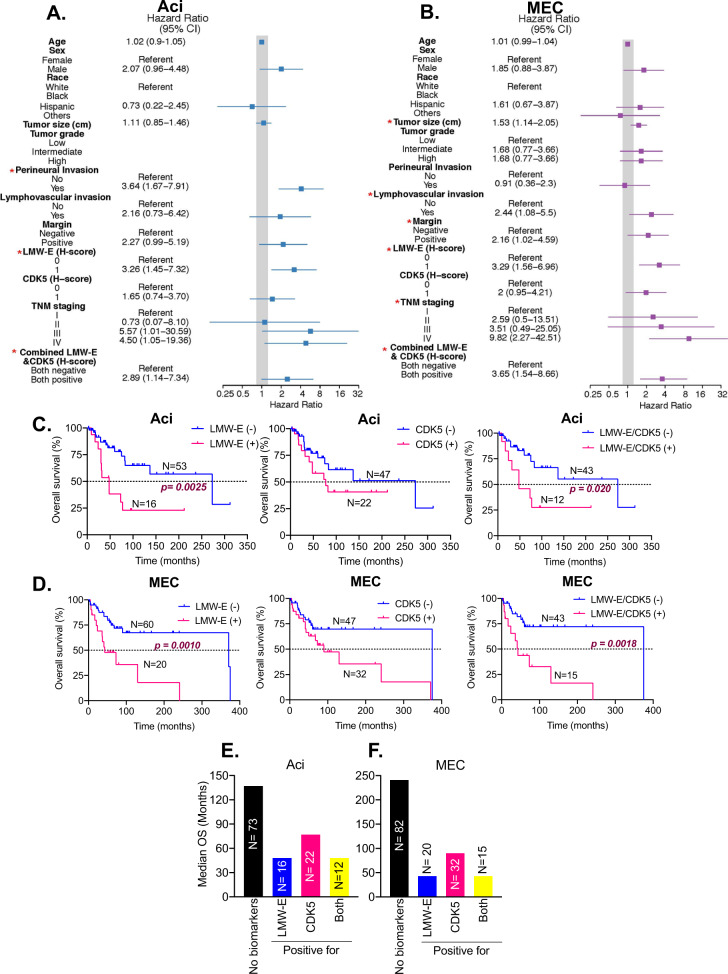
High cyclin E and CDK5 expression can predict overall survival in Aci and MEC subtypes of salivary gland tumors. **A**, **B** Forest plots analysis indicating univariable analysis of the indicated variables with overall survival in each Aci and MEC subtypes. The solid gray line indicates a hazard ratio of 1 for the overall population. Variables with *p* values < 0.05 are marked with * and are significant predictors of recurrence-free survival for each subtype, calculated using the Cox proportional-hazards model. **C**, **D** Kaplan–Meier plots for subtypes Aci and MEC respectively, according to LMW-E, CDK5, or LMW-E/CDK5 expression. The expression of LMW-E and CDK5, individually and combined, was significantly associated with decreased overall survival in the indicated subtypes. *p* Values indicate comparison between survival curves, calculated using the log-rank (Mantel–Cox) test. **E**, **F** Graph comparing median OS without any biomarkers (black bars) to that when patient tumor samples are positive (*H*-score = 1) for LMW-E (blue bars), CDK5 (pink bars), or LMW-E /CDK5 (yellow bars); for each subtypes Aci and MEC.

We also assessed if LMW-E and CDK5 co-overexpression are prognosticators of the poor overall survival (OS). When assessing OS, the univariable analysis indicated that LMW-E and CDK5 co-expression was predictive of the poor OS only for subtypes Aci (HR = 2.89) and MEC (HR = 3.65) (Fig. 7A, B and Supplementary Table 5). Individual high LMW-E expression was significantly associated with poor OS for subtypes Aci and MEC with HR ratios of 3.26 and 3.29, respectively. Subtypes AdCC and SDC showed no association with OS in the univariable analysis (Supplementary Fig. 6A, B). The median OS in each subtype revealed that only LMW-E (and not CDK5) expression impacted survival in subtypes Aci (48 months vs. 273 months) and MEC (43 vs. 371 months) (Fig. 7C, D, Supplementary Table 6). LMW-E and CDK5 had no impact on the median survival of the AdCC and SDC subtypes (Supplementary Fig. 6C–F). Thus, LMW-E and CDK5 are associated with a decline in the OS of patients with the Aci and MEC subtypes (Fig. 7E, F).

Lastly, we constructed a multivariable model with all the significant markers from our univariable analysis (those marked by an asterisk in Fig. 5 and Supplementary Table 3). These analyses revealed that high LMW-E expression is an independent biomarker associated with poor RFS in subtypes AdCC (HR = 1.78, *p* = 0.0217) and MEC (HR = 5.68, *p* = 0.0039) (Supplementary Table 7) and poor OS for Aci patients alone (HR = 3.44, *p* = 0.0448) (Supplementary Table 8). High CDK5 expression predicted poor RFS only in AdCC patients (HR = 1.78, *p* = 0.0268) in the multivariable analysis model (Supplementary Table 7). Collectively, the univariable and multivariable analyses indicate that high LMW-E and CDK5 expression are significant predictors of poor RFS in patients, independent of the histological subtype of the salivary tumors.

## Discussion

In this study, we report LMW-E and CDK5 as novel and targetable biomarkers of SGCs that can predict recurrence for its four major subtypes (MEC, AdCC, Aci, and SDC). We provide evidence for a novel transgenic mouse model of SGC representing the main salivary gland tumor subtypes. Our preclinical studies suggest that the LMW-E/CDK5 axis is a viable therapeutic target, providing the rationale for translation of CDK5 inhibitors for future in vivo and clinical studies. Lastly, our clinical study sets the precedent for evaluating LMW-E and CDK5 status in SGC patients for stratifying those patients (i.e., LMW-E/CDK5 high) that are likely to respond to CDK5 inhibitors.

Full-length cyclin E is a key cell cycle regulator with multiple functions including the activation of CDK2 and the maintenance of genome integrity. However, when cleaved to its LMW-E isoforms^28^, cyclin E is oncogenic and a predictive marker of recurrence in breast cancer patients^13–15,27^. To interrogate the role of LMW-E in breast cancer, we developed a transgenic mouse model LMW-E-T1; p53+/−, which primarily gives rise to mammary gland tumors^29^. Serendipitously, we found that these mice developed tumors in the salivary gland with 25% frequency, even in the absence of CDK2, the primary binding partner of cyclin E^17^. A key advantage of the LMW-E-T1; p53+/− model characterized here is that the mice develop intermediate to high-grade adenocarcinomas in the parotid gland, independent of subtype and thus recapitulating the majority of SGCs observed clinically. Comparing this to the existing mouse models (Supplementary Table 9), we observe that the mouse models are either restricted to a particular subtype (Ela-Cre-ERT-LGL KRAS model of SDC^8^) or that they do not recapitulate the clinical features of SGTs (MMTV APC-/- Pten-/- a model of Aci and MMTV- RANKL model of poorly differentiated adenocarcinomas^9,30^). When comparing SGC frequency, the Ela-Cre-ERT-LGL KRAS model of SDC has the advantage of having a 100% frequency of tumor induction. The MMTV APC−/− Pten−/− model of Aci is comparable to the transgenic model in our study showing SGC frequencies of 23% and 25%, respectively. This limitation of low SGC frequency (25%) vs. a high frequency of mammary tumors in the MMTV-LMW-E-T1; p53+/ model reported here, will be addressed in future studies where we will adopt the hybrid MMTV-Cre-Rosa 26 background mice, which show a reduced frequency of mammary tumors^30^ and increased SGC frequency. For the purposes of the current study, we focused our efforts on understanding of the role of LMW-E in salivary gland tumors and identifying the key CDK(s) aiding in salivary gland tumorigenesis.

CDK2 and CDK1 are known to be the functionally redundant partners of LMW-E, which can drive tumorigenesis^19,31^. However, our study demonstrated the presence of a third kinase—CDK5 as a key LMW-E associated CDK that can substitute for CDK2 and drive LMW-E dependent salivary gland tumorigenesis. These findings of CDK5 being a driver in SGCs is not surprising, given that CDK5 shares 60% similarity with the amino acid sequences of CDK1 and CDK2^32^. Historically, CDK5 has been known for its non-cell cycle roles in neuronal and non-neuronal cells^32^ and is thus an important therapeutic target for different neurodegenerative diseases^33^.

The clinical data presented in this report provides the rationale for the IHC assessment of LMW-E and CDK5 alongside standard staging parameters; to predict recurrence in SGC patients. We show that LMW-E and CDK5 are concordantly expressed (both positive or both negative) in 70% of SGC patients, independent of histological subtype and their co-expression predicts poor RFS. Prior genomic and molecular studies in SGC^34–37^ have been insufficient in identifying biomarkers that could predict RFS in multiple histologic subtypes. High LMW-E expression is also an independent prognostic marker of poor RFS in MEC and AdCC and poor OS in the Aci subtype of patients, respectively, as indicated by our multivariable analysis. When presented as intermediate to high-grade tumors, both MEC and AdCC are aggressive subtypes, where patients present with a high rate of recurrence and distant metastases^2^. In the present study, 81% of MEC patients and 85% AdCC patients presented with intermediate to high-grade tumors. The subsequent finding of high LMW-E expression and its correlation with RFS in these two subtypes of SGC was thus consistent with the intermediate to high-grade tumors in these patients. Aci, on the other hand is a relatively indolent and slow-growing tumor, a subset of which genetically resembles ductal breast carcinoma, in which the prognostic value of LMW-E has been well-established^13–15,27^.

## Materials and methods

Generation of transgenic mouse models and stable cell lines, affinity purification, and IHC analysis are included in Supplemental Methods.

### Patient population

The retrospective collection of clinical samples was approved by The University of Texas MD Anderson Cancer Center IRB and patients signed consent for participation. Patient 18 years of age or older, with a diagnosis of clinical stage I–IV salivary gland cancer, were eligible for enrollment and all signed the front door and study-specific consent forms. The stage was based on the eighth edition of the American Joint Committee on Cancer staging criteria^38^. Clinical data included patient demographics, tumor characteristics, clinical subtypes, clinical-stage, pathologic stage, recurrence, and survival collected for each patient. Study endpoints were overall survival (OS) and recurrence-free survival (RFS), as a function of LMW-E and CDK5 levels. The patient’s age ranged from 22 to 97 years (mean age 63 years). Totally, 50.8% patients of all patients were female and 48.5% were males. In total, 58.8% of the patient population presented with clinical TNM staging IV, with a comparable number of patients showing absence (43.6%) and presence (50.3%) of perineural invasion (PNI). Of the 482 patients, a subtype of adenoid cystic carcinoma (AdCC) comprised 44.6% cases followed by SDC comprising 21.7%. Comparable (16.6% and 17.1%, respectively) number of acinic cell carcinoma (Aci) and mucoepidermoid carcinoma (MEC) cases were observed in the patient population.

### Scoring of salivary tumor sections

Cyclin E and CDK5 scoring was performed by three pathologists blinded to patient outcomes. Cyclin E nuclear and cytoplasmic staining scores were independently assigned according to their staining intensity and as previously described^14,15,24–27^. For both cyclin E and CDK5, >50% nuclear or cytoplasmic positivity was considered positive. Representative IHC images for both cyclin E and CDK5 are presented in Fig. 4B.

### Cyclin E scoring

Nuclear and cytoplasmic staining scores were independently assigned according to the percentage of cells stained (>50%) and their staining intensity (1 = no staining, 2 = weak staining, 3 = intermediate staining, and 4 = strong staining). The nuclear and cytoplasmic scores were combined to generate the four cyclin E immunophenotypes. Phenotype 1 indicates no nuclear and no cytoplasmic staining; phenotype 2, positive nuclear staining and no or weak cytoplasmic staining; phenotype 3, positive nuclear and positive cytoplasmic staining; phenotype 4, positive cytoplasmic and no or weak nuclear staining. LMW-E status was then assigned as follows: LMW-E negative (those that were scored as phenotypes 1 or 2—no staining or just nuclear staining). LMW-E positive (those that were scored as phenotypes 3 or 4—nuclear + cytoplasmic or just cytoplasmic staining).

### CDK5 staining

Nuclear and cytoplasmic scores for CDK5 were also independently assigned according to the percentage of cells stained (>50%) and their staining intensity: (1 = low, 2 = medium, and 3 = high). The extent of staining was classified as the percentage of cells with CDK5-positive nuclei or cytoplasm on a scale of 0 (<50%) to 1 (>50%). The final immunoreactivity score was determined by multiplying the intensity score (1, 2, or 3) by the percent staining extent score (0 or 1), with a minimum score of 0 and a maximum score of 3. Cytoplasmic scores of ≥2 indicated positivity for CDK5 and translated to an *H* score of 1. While scores less than 2, translated to an *H* score of 0 and indicated cytoplasmic CDK5 negativity.

### Statistical analysis

The patient population described in Table 1 was subjected to a test of homogeneity of each subtype, one-way ANOVA, and chi-squared tests to compare distributions of continuous and categorical factors between individual subtype groups and as compared with other subtypes (pooled). Kaplan–Meier curves generated estimate RFS and OS, were compared using log-rank (Mantel–Cox) test, with *p* ≤ 0.05 being significant. For univariable analysis of OS and RFS for all factors was performed using the Cox-proportional hazards model. For multivariable Cox proportional hazards modeling of OS and RFS for all subtype groups, all factors from Table 1 were considered, except for those with missing values. The HRs (95% CI) for stage II compared to stage I was 1.71 (0.24–12.37), 0.39 (0.10–1.46), 6.09 (0.73–50.69), for Aci, ADCC, MEC groups, respectively for RFS (stage II HR not estimated for SDC due to small sample size in that category) (Supplementary Table 3). For OS, the HRs (95% CI) for stage II compared to stage I was 0.73 (0.07–8.10), 1.32 (0.38–4.65), 2.59 (0.5–13.51) for Aci, ADCC, and MEC groups (stage II HR was not estimated for SDC group due to small sample size in that category) (Supplementary Table 5). Based on the 95% CIs, there was no evidence that HR between stages I and II were different. Hence, in multivariable analyses, we combined stages I and II for all the subgroups.

## Supplementary information

Supplemental Methods

Supplementary tables 1-9

Supplementary Figures and Figures legends
