# Supplementary material for: LMW cyclin E and its novel catalytic partner CDK5 are therapeutic targets and prognostic biomarkers in salivary gland cancers
Source: Oncogenesis. 2021 May 14;10(5):40. doi: 10.1038/s41389-021-00324-z (PMC8121779; doi:10.1038/s41389-021-00324-z)
Supplement: Supplementary file 1 — Supplemental Methods [file 41389_2021_324_MOESM1_ESM.pdf]

Supplemental Methods

LMW cyclin E and its novel catalytic partner CDK5 are therapeutic targets and prognostic biomarkers in salivary gland cancers

Amriti R. Lulla[1*], Said Akli[1*], Cansu Karakas[1#], Min Jin Ha[2#], Natalie W. Fowlkes[3], Yoshitsugu Mitani[4], Tuyen Bui[1], Jing Wang[5], Xiayu Rao[5], Kelly K. Hunt[6], Laurent Meijer[7,] Adel K. El-Naggar[4] and Khandan Keyomarsi[1+]

Generation and analysis of transgenic mice.

CDK2-deficient mice (1), p53-deficent mice (2), and MMTV-LMW-E transgenic mice (3), all female FVB/NJ, age 1-16 months were generated as previously described. The breeding strategy to generate the triple transgenic mice is described in (4). Monitoring for tumor development was performed biweekly. Mice were humanely euthanized when tumors reached a diameter of 1.5 cm. All tissues collected divided for IHC in 10% neutral buffered formalin and RNA and protein by snap freezing. Histological analysis of tumor tissue was done by hematoxylin and eosin (H&E) staining and evaluated by a veterinary pathologist according to INHAND criteria (5) on a Leica DM 2500 light microscope. Histologic features of tumors were reported, and representative images were captured using a Leica Microsystems DFC 495 digital camera. All procedures were performed per approved IACUC procedures.

Immunohistochemistry

Immunohistochemical analysis of cyclin E were performed as previously described (27), with the following modifications for CDK5 staining. 5 μm-thick slide sections were obtained from formalin-

fixed, paraffin-embedded blocks and stained for antibodies against cyclin E and CDK5. Briefly, slide sections were deparaffinized in xylene and hydrated in a series of graded alcohol dilutions, followed by boiling for 20 minutes in 10 mM sodium citrate (pH 6.5) for antigen retrieval and subsequently cooled at room temperature for 30 minutes. 3% hydrogen peroxide used to block endogenous peroxidase activity. Sections were incubated overnight and 1 hour at room temperature with primary antibodies against cyclin E (rabbit polyclonal, Catalog # sc-198; Santa Cruz, 1:1000 dilution), and with CDK5 (rabbit polyclonal, Catalog # sc-6247; Santa Cruz, 1:500 dilution), respectively. After the sections were rinsed, antibodies were detected with a secondary antibody from the Vectastain Elite ABC Kit (PK6101 and PK6102; Vector Laboratories, Burlingame, CA, USA). Color development was performed using 3,3-diaminobenzidine. Counterstaining was provided by staining with hematoxylin. All washing steps were performed in PBS alone and PBS with 0.1% Tween.

Establishment of stable cell lines and affinity purification of S protein-FLAG-Streptavidin binding protein (SFB)-tagged cyclin E-LMW and SFB tagged cyclin E-LMW^{R130A} complexes. HSG cell lines were derived as described in (6) and were authenticated by MD Anderson Cancer Center's Cell Line Authentication core facility and were free of mycoplasma (assessed every 6 months). HSG cells were transfected with plasmids encoding SFB-tagged cyclin E-LMW and SFB-tagged cyclin E-LMW^{R103A}, followed by selection with 2 μg/ml puromycin. For affinity purification (all procedures done at 4°C), HSG cells were lysed in NETN buffer (NaCl; Tris-Cl; EDTA; Nonident P-40) for 20 minutes. Crude lysates were centrifuged at 14,000 rpm for 20 minutes. Supernatants were incubated with streptavidin-conjugated beads (Amersham) for 2 hours, followed by washing three times with NETN buffer, and bound proteins were eluted with NETN buffer containing 2 mg/ml biotin (Sigma) for 2 hours. Eluents were incubated with S protein beads (Novagen) for 1 hour, following washing three times with NETN buffer and subjected to

SDS-PAGE. Protein bands were excised and subjected to mass spectrometry analysis (performed by Taplin Mass Spectrometry Facility, Harvard Medical School).

shRNA and siRNA knockdown

shRNA and siRNA studies were performed as previously described (7). Briefly, CDK2 shRNA constructs were purchased from Thermo Scientific (shCDK2 clones 41 and 42) and Dharmacon (shCDK2 clones 569 and 573). pGIPZ vector scrambled shRNA was purchased from Dharmacon. To generate lentivirus expressing shRNA, HEK293T cells were co-transfected with pCMVdeltaR8.2, pMD2.G pGIPZ vector and either scrambled shRNA or shRNA against the gene of interest using Lipofectamine 3000 reagent according to the manufacturer's protocol. After 72 hours of transfection, the virus-containing medium was collected, filtered through a 0.45-μm filter, and added to the cells of interest in the presence of 8 μg/mL of polybrene (Millipore). GFP expression was confirmed and the lentivirus-infected cells were either GFP sorted and/or selected with 1 μg/mL puromycin (InvivoGen, San Diego, CA).

For siRNA knockdown studies of CDK5 and CDK2, 0.1 x 10^6 cells were transfected with either scramble duplex (SCR, ON-TARGETplus, D-001810-10-05) or CDK5 siRNA (Thermo Fisher Scientific, Catalog # AM51331) or CDK2 siRNA (SantaCruz Biotechnology, Catalog # sc-29259) to a net concentration of 50 nM using Lipofectamine 3000. Transfected cells were cultured for 72 hours and used for colony formation assays and cell cycle analysis, described in the Supplementary methods.

Western blot analysis, immunoprecipitation, immunodepletion and kinase assays.

Protein expression using murine and human tumor tissues and cell lines were analyzed by western blot analysis as previously described (3, 4, 8, 9). Briefly, tumor tissues were

homogenized and lysed in RIPA buffer, examined for total protein and subjected to western blot analysis with following antibodies: Cyclin E (HE12; sc247), CDK2 (D-12; sc6248), CDK1 (17, sc-54), p27 (C-19, sc-528), CDK5 (C-8, sc-173) PARP (CST #9542) and β-actin (C4; MAB1501). For immunoprecipitation studies, 250-400 μg of tissue extracts/cell lysates was used per immunoprecipitation with polyclonal antibody to cyclin E coupled to protein A beads or monoclonal antibody to Flag, or CDK1 coupled to protein G beads. For immunodepletion of CDK1, 250μg of salivary gland tumor lysates were incubated with 1μg of CDK1 antibody coupled to protein G beads overnight at 4°C. After centrifugation, the supernatant was incubated with 1μg of CDK1 antibody coupled to protein G beads for one hour at 4°C for 2 rounds. The last supernatant depleted of CDK1 was used for immunoprecipitation with cyclin E followed by histone H1 kinase assay. For kinase assays with HH1 and GST-Rb, the immunoprecipitates were incubated with kinase assay buffer containing 60 mmol/L cold ATP, 5 mCi of [32P] ATP, and 5mg of histone H1 (Roche Diagnostics Corporation, Basel, Switzerland) or 1 µg of GST-Rb (SantaCruz Biotechnology), in a final volume of 30µL at 37°C for 30 minutes. The products of the reaction were analyzed on 13% SDS-PAGE gels, and the gels were stained, destained, dried, and exposed to X-ray film. For quantitation, the protein bands corresponding to histone H1 and GST-Rb were excised, and the radioactivity of each band was measured by Cerenkov counting.

Cell viability and cell proliferation assays

Cell viability and proliferation assays were performed as previously described (7) with the following modifications. Briefly, a total of 500-1000 cells/ well were plated in 96-well plates, followed by treatment with SNS-032 at 0-1μM, Ro-3306 at 0-10μM and P Palbociclib at 0-10μM. Media was replenished every 3 days. Cell viability was measured 12-days post treatment using Cell-Titer-Blue® Fluorescent Cell Viability Assay (Promega, Madison, WI, USA). The percent-cell viability was calculated by normalizing the fluorescence signal to DMSO treated wells. All

treatments were performed in triplicates and reported as % viability ± SEM, compared to DMSO control. GraphPad prism was used to generate dose response curves and calculate EC_{50} values.

Colony forming assays

Colony formation assays were performed as previously described (7) with the following modifications. For colony formation post siRNA transfection, a total of 0.1 x 10^6 cells were transfected with either scramble duplex (SCR) or CDK5 siRNA to net concentration of 50 nM for 72 hours. For colony formation post drug treatments, a total of 0.1 x 10^6 cells were treated with the indicated concentration of drug. At 72 hours, transfected/ drug- treated cells were harvested and 500 cells per group were plated in triplicate in 6-well plates for colony formation. Colonies were stained with 0.25% crystal violet on Day 10, imaged, counted and reported as % colony formation ± SEM, normalized to SCR for each cell line. All experiments were repeated three times (3 biological replicates). Statistically significant differences were calculated using the unpaired Student's t-test with Welch's correction, with the reference groups being SCR (for siRNA transfection experiments) and no treatment (for drug treatment experiments) respectively.

Cell cycle analysis

Cell cycle analysis were performed as previously described (10) with the following modifications. Briefly, a total of 0.1 x 10^6 cells were transfected with either scramble duplex (SCR) or CDK5 siRNA to net concentration of 50 nM. 72 hours (3 days) post transfection, cells were harvested by trypsinization, washed with 1X PBS and fixed in 70% ethanol at 4°C overnight. Cells were then stained with propidium iodide (1 mg/mL) in FACS buffer [composed of RNaseA (1 mg/mL), 0.1% (v/v) Triton X-100 (Sigma), 1% fetal bovine serum (FBS), all added in 1X PBS] at 4 °C overnight. The Beckman Coulter Gallios 560 flow cytometer (Beckman Coulter, Brea, CA, USA) was used for data collection, followed by data analysis using FlowJo software.

References:

1. Ortega S, Prieto I, Odajima J, Martin A, Dubus P, Sotillo R, et al. Cyclin-dependent kinase 2 is essential for meiosis but not for mitotic cell division in mice. Nature genetics. 2003;35(1):25-31.
2. Donehower LA, Harvey M, Slagle BL, McArthur MJ, Montgomery CA, Jr., Butel JS, et al. Mice deficient for p53 are developmentally normal but susceptible to spontaneous tumours. Nature. 1992;356(6366):215-21.
3. Akli S, Van Pelt CS, Bui T, Multani AS, Chang S, Johnson D, et al. Overexpression of the low molecular weight cyclin E in transgenic mice induces metastatic mammary carcinomas through the disruption of the ARF-p53 pathway. Cancer Res. 2007;67(15):7212-22.
4. Akli S, Van Pelt CS, Bui T, Meijer L, Keyomarsi K. Cdk2 is required for breast cancer mediated by the low-molecular-weight isoform of cyclin E. Cancer Res. 2011;71(9):3377-86.
5. Nolte T, Brander-Weber P, Dangler C, Deschl U, Elwell MR, Greaves P, et al. Nonproliferative and Proliferative Lesions of the Gastrointestinal Tract, Pancreas and Salivary Glands of the Rat and Mouse. J Toxicol Pathol. 2016;29(1 Suppl):1S-125S.
6. Mitani Y, Li J, Weber RS, Lippman SL, Flores ER, Caulin C, et al. Expression and regulation of the DeltaN and TAp63 isoforms in salivary gland tumorigenesis clinical and experimental findings. Am J Pathol. 2011;179(1):391-9.
7. Carey JPW, Karakas C, Bui T, Chen X, Vijayaraghavan S, Zhao Y, et al. Synthetic Lethality of PARP Inhibitors in Combination with MYC Blockade Is Independent of BRCA Status in Triple-Negative Breast Cancer. Cancer Res. 2018;78(3):742-57.
8. Akli S, Bui T, Wingate H, Biernacka A, Moulder S, Tucker SL, et al. Low-molecular-weight cyclin E can bypass letrozole-induced G1 arrest in human breast cancer cells and tumors. Clin Cancer Res. 2010;16(4):1179-90.
9. Keyomarsi K, Tucker SL, Buchholz TA, Callister M, Ding Y, Hortobagyi GN, et al. Cyclin E and survival in patients with breast cancer. N Engl J Med. 2002;347(20):1566-75.
10. Francis AM, Alexander A, Liu Y, Vijayaraghavan S, Low KH, Yang D, et al. CDK4/6 Inhibitors Sensitize Rb-positive Sarcoma Cells to Wee1 Kinase Inhibition through Reversible Cell-Cycle Arrest. Mol Cancer Ther. 2017;16(9):1751-64.
