# Supplementary material for: LMW cyclin E and its novel catalytic partner CDK5 are therapeutic targets and prognostic biomarkers in salivary gland cancers
Source: Oncogenesis. 2021 May 14;10(5):40. doi: 10.1038/s41389-021-00324-z (PMC8121779; doi:10.1038/s41389-021-00324-z)
Supplement: Supplementary file 2 — Supplementary tables 1-9 [file 41389_2021_324_MOESM2_ESM.pdf]

Case #	Label	Site of origin	Histological subtype*	LMW-E expression
**1**	338 C5	Parotid	MEC	No
**2**	364 B2	Tongue, NOS	Aci	No
**3**	407 A3	Parotid	SC	Yes
**4**	431 E4	Lymph node, neck	Aci	Yes
**5**	438 E7	Parotid	AC	Yes
**6**	461 E5	Parotid	UnC	Yes
**7**	467 D8	Parotid	CexPA	Yes
**8**	457 H7	Parotid	Aci	No
**9**	332 F6	Parotid	CexPA	Yes
**10**	297 H1	Parotid	Aci	No
**11**	245 D7	Parotid	PA	Yes
**12**	400 A3	Submandibular	RMS	Yes
**13**	386 B1	Parotid	CexPA	Yes
**14**	385 D5	Floor of mouth	Aci	Yes
**15**	317 D1	Larynx	Aci	No
**16**	292 F3	Thyroid, NOS	MEC	No
**17**	429 C2	Orbital	Aci	Yes
**18**	430 A4	Parotid	SDC	No

Supplementary Table 1. Summary of tumor sample characteristics, obtained from salivary gland cancer patients. A list of 18 salivary gland tumor samples from patients, indicating the site of tumor origin, their clinical histological classification and status of LMW-E expression status analyzed western blot analysis.

***Abbreviations:**

MEC, mucoepidermoid carcinoma
Aci, adenoid cystic carcinoma
SC, sebaceous carcinoma
AC, adenocarcinoma
UnC, undifferentiated carcinoma
CexPA, carcinoma ex-pleomorphic adenoma
PA, pleomorphic adenoma
RMS, rhabdomyosarcoma
SDC, salivary duct carcinoma.

Primary tumor site	Aci (N=80)	AdCC (N=215)	MEC (N=82)	SDC (N=105)
Parotid gland	74 (92.5)	31 (14.4)	27 (32.9)	82 (78.1)
Submandibular gland	2 (2.5)	21 (9.8)	3 (3.7)	8 (7.6)
Sublingual gland	0 (0)	2 (0.9)	1 (1.2)	0 (0)
Maxilla and maxillary sinuses	0 (0)	56 (26.0)	10 (12.2)	4 (3.8)
Palate	0 (0)	17 (7.9)	5 (6.1)	0 (0)
Orbit	0 (0)	16 (7.4)	0 (0)	1 (1.0)
Tongue	0 (0)	14 (6.5)	14 (17.1)	2 (1.9)
Floor of mouth	0 (0)	10 (4.7)	0 (0)	0 (0)
Mandible	0 (0)	8 (3.7)	8 (9.8)	3 (2.9)
Buccal mucosa	0 (0)	6 (2.8)	1 (1.2)	1 (1.0)
Nasal cavity	0 (0)	6 (2.8)	0 (0)	2 (1.9)
Trachea	0 (0)	4 (1.9)	1 (1.2)	0 (0)
Lung	0 (0)	0 (0)	4 (4.9)	0 (0)
Ethmoid sinus	0 (0)	3 (1.4)	0 (0)	0 (0)
Lacrimal gland	0 (0)	3 (1.4)	0 (0)	0 (0)
Larynx	0 (0)	3 (1.4)	0 (0)	0 (0)
Pharynx	0 (0)	3 (1.4)	0 (0)	0 (0)
Laryngopharynx	0 (0)	2 (0.9)	0 (0)	0 (0)
Nasopharynx	0 (0)	2 (0.9)	1 (1.2)	0 (0)
Scalp	0 (0)	3 (1.4)	1 (1.2)	0 (0)
Parapharyngeal	2 (2.5)	0 (0)	0 (0)	0 (0)
Unknown	2 (2.5)	0 (0)	1 (1.2)	0 (0)
Ear	0 (0)	1 (0.5)	0 (0)	0 (0)
Lip	0 (0)	1 (0.5)	1 (1.2)	0 (0)
Retromolar trigone	0 (0)	1 (0.5)	2 (2.4)	0 (0)
Skull; NOS	0 (0)	1 (0.5)	0 (0)	0 (0)
Sphenoid sinus	0 (0)	1 (0.5)	0 (0)	0 (0)
Trachea	0 (0)	0 (0)	1 (1.2)	0 (0)
Thyroid, NOS	0 (0)	0 (0)	1 (1.2)	0 (0)
Oropharynx	0 (0)	0 (0)	1 (1.2)	0 (0)
Gingiva	0 (0)	0 (0)	0 (0)	1 (0.9)
Neck	0 (0)	0 (0)	0 (0)	1 (0.9)
**Number in bracket () indicate % frequency**				

Supplementary Table 2. Primary tumor site with frequency of occurrence: Summary of the primary sites for each subtype. Number in the bracket (), shows the percent frequency of occurrence at the indicated site. The % frequency was calculated as a function of the total number of tumors in each subtype. Grey shaded rows indicate sites with the highest frequencies in each subtype, mainly observed in the major salivary glands and in the maxilla.

Univariable analysis for Recurrence-Free Survival in each subype								
Variable	Aci		ADCC		MEC		SDC	
	HR (95% CI)	P-value	HR (95% CI)	P-value	HR (95% CI)	P-value	HR (95% CI)	P-value
**Age (years)**	1 (0.98-1.02)	0.8350	0.98 (0.96-0.99)	0.0007	1.01 (0.99-1.03)	0.4857	0.99 (0.97-1.02)	0.6641
**Sex**								
Female	Referent	0.0523	Referent	0.2109	Referent	0.5107	Referent	0.3407
Male	1.98 (0.99-3.94)		1.27 (0.87-1.86)		1.28 (0.61-2.66)		1.32 (0.75-2.34)	
**Race**								
White	Referent	0.6838*	Referent	0.8878	Referent	0.8797*	Referent	0.1278
Black			1.21 (0.61-2.4)				3.43 (1.22-9.69)	
Hispanic	0.69 (0.24-1.98)		1.19 (0.68-2.06)		0.85 (0.32-2.23)		1.4 (0.43-4.52)	
Others	0.56 (0.08-4.1)		0.9 (0.29-2.87)		0.74 (0.17-3.15)		1.55 (0.21-11.32)	
**Tumor size (CM)**	1.01 (0.77-1.31)	0.9503	1.15 (1.02-1.29)	0.0221	1.34 (1.01-1.78)	0.0408	0.99 (0.86-1.14)	0.9410
**Tumor grade**								
Low			Referent	0.2551	Referent	0.0254	Referent	0.0188
Intermediate			1.42 (0.78-2.58)		2.3 (1.11-4.76)		5.52 (1.33-22.94)	
High			1.42 (0.78-2.58)		2.3 (1.11-4.76)		5.52 (1.33-22.94)	
**Perineural invasion**								
No	Referent	0.0037	Referent	0.2486	Referent	0.8328	Referent	0.1449
Yes	3.03 (1.43-6.39)		1.31 (0.83-2.06)		1.1 (0.47-2.56)		1.53 (0.86-2.72)	
**Lymphovascular invasion**								
No	Referent	0.2409	Referent	0.0065	Referent	0.0013	Referent	0.0128
Yes	1.78 (0.68-4.64)		1.99 (1.21-3.25)		3.5 (1.63-7.51)		2 (1.16-3.45)	
**Margin**								
Negative	Referent	0.0007	Referent	0.0449	Referent	0.0144	Referent	0.0778
Positive	3.97 (1.78-8.84)		1.48 (1.01-2.16)		2.5 (1.2-5.2)		1.81 (0.94-3.48)	
**LMW-E H-score**								
0	Referent	0.0006	Referent	<0.0001	Referent	<0.0001	Referent	0.0084
1	3.46 (1.71-7.01)		2.51 (1.66-3.79)		6.54 (3.09-13.81)		2.24 (1.23-4.08)	
**CDK5 H-score**								
0	Referent	0.0001	Referent	<0.0001	Referent	0.0191	Referent	0.0093
1	3.98 (1.98-8.01)		2.58 (1.67-3.97)		2.48 (1.16-5.3)		2.15 (1.21-3.83)	
**Clinical TNM stage**								
I	Referent	0.0024	Referent	<0.0001	Referent	0.0003	Referent	0.0015**
II	1.71 (0.24-12.37)		0.39 (0.1-1.46)		6.09 (0.73-50.69)			
III	3.79 (0.61-23.54)		0.24 (0.05-1.08)		5.7 (0.51-63.87)		2.28 (0.32-16.19)	
IV	9.64 (2.23-41.64)		2.76 (1.01-7.56)		25.79 (3.43-193.62)		9.66 (2.32-40.3)	
**Combined LMW-E & CDK5 H-score**								
Both negative	Referent	0.0002	Referent	<0.0001	Referent	<0.0001	Referent	0.0032
Both positive	5.08 (2.15-12.02)		4.05 (2.33-7.04)		6.79 (2.74-16.83)		3.26 (1.49-7.15)	

* Black category is combined into Others category

** Combined I and II

Cox model fitting did not work for small sample size for tumor grade in Aci subtype

Supplementary Table 3. Univariable analysis for RFS using variables LMW-E/CDK5 expression along with standard markers of clinical outcome

Tabular representation of univariable analysis of the indicated variables with recurrence-free survival in each subtype. Variables with p-values <0.05 indicate predictors of recurrence- free survival for each subtype, calculated using the Cox proportional-hazards model.

Recurrence-free survival	Aci		ADCC		MEC		SDC	
**Variable**	**n**	**Median Survival**	**n**	**Median Survival**	**n**	**Median Survival**	**n**	**Median Survival**
**No biomarkers**	73	78	203	90	82	127	99	33
**LMW-E H-score**								
0	53	138	107	161	60	296	38	NA
1	16	18	65	43	20	11.5	54	19
**CDK5 H-score**								
0	47	203	90	161	47	NA	40	85
1	22	16	84	47	32	62	52	17
**Combined LMW-E & CDK5 H-score**								
Both negative	43	203	70	NA	43	NA	28	NA
Both positive	12	13.5	45	30	15	14	42	17
**Median survival is in months**								

Supplementary Table 4. Median RFS as function of no biomarker or differential (+ or -) LMW-E and CDK5 expression. Summary of median RFS focused on variables of no biomarker, LMW-E, CDK5 or LMW-E/CDK5 low (H-score =0) and high expression (H-score =1).

Univariable analysis for Overall Survival in each subype								
**Variable**	**Aci**		**ADCC**		**MEC**		**SDC**	
	**HR (95% CI)**	**P-value**	**HR (95% CI)**	**P-value**	**HR (95% CI)**	**P-value**	**HR (95% CI)**	**P-value**
**Age (years)**	1.02 (0.99-1.05)	0.1485	0.99 (0.98-1)	0.2303	1.01 (0.99-1.04)	0.2054	0.99 (0.98-1.01)	0.5536
**Sex**								
Female	Referent	0.0639	Referent	0.6314	Referent	0.1050	Referent	0.9689
Male	2.07 (0.96-4.48)		0.92 (0.65-1.3)		1.85 (0.88-3.87)		0.99 (0.6-1.62)	
**Race**								
White	Referent	0.6129	Referent	0.5415	Referent	0.2825	Referent	0.0190
Black			1.08 (0.58-1.99)				5.32 (1.85-15.29)	
Hispanic	0.73 (0.22-2.45)		1.09 (0.65-1.82)		1.61 (0.67-3.87)		1.59 (0.49-5.09)	
Others			0.24 (0.03-1.75)				1.07 (0.15-7.76)	
**Tumor size (CM)**	1.11 (0.85-1.46)	0.4266	1.09 (0.94-1.26)	0.2479	1.53 (1.14-2.05)	0.0043	1.02 (0.89-1.16)	0.8118
**Tumor grade**								
Low			Referent	0.0139	Referent	0.1940	Referent	0.8970
Intermediate			1.95 (1.15-3.31)		1.68 (0.77-3.66)		1.06 (0.45-2.49)	
High			1.95 (1.15-3.31)		1.68 (0.77-3.66)		1.06 (0.45-2.49)	
**Perineural invasion**								
No	Referent	0.0011	Referent	0.3012	Referent	0.8482	Referent	0.3051
Yes	3.64 (1.67-7.91)		1.24 (0.82-1.88)		0.91 (0.36-2.3)		1.3 (0.78-2.17)	
**Lymphovascular invasion**								
No	Referent	0.1637	Referent	0.0049	Referent	0.0321	Referent	0.0367
Yes	2.16 (0.73-6.42)		1.96 (1.23-3.14)		2.44 (1.08-5.5)		1.68 (1.03-2.72)	
**Margin**								
Negative	Referent	0.0530	Referent	0.2461	Referent	0.0452	Referent	0.0949
Positive	2.27 (0.99-5.19)		1.23 (0.86-1.76)		2.16 (1.02-4.59)		1.6 (0.92-2.77)	
**LMW-E H-score**								
0	Referent	0.0041	Referent	0.6103	Referent	0.0018	Referent	0.5912
1	3.26 (1.45-7.32)		1.11 (0.75-1.64)		3.29 (1.56-6.96)		1.16 (0.68-1.96)	
**CDK5 H-score**								
0	Referent	0.2232	Referent	0.2997	Referent	0.0667	Referent	0.4143
1	1.65 (0.74-3.7)		1.23 (0.83-1.81)		2 (0.95-4.21)		1.24 (0.74-2.06)	
**Clinical TNM stage**								
I	Referent	0.0609	Referent	0.0091	Referent	0.0019	Referent	0.0308*
II	0.73 (0.07-8.1)		1.32 (0.38-4.65)		2.59 (0.5-13.51)			
III	5.57 (1.01-30.59)		1.09 (0.3-3.91)		3.51 (0.49-25.05)		1.24 (0.39-3.92)	
IV	4.5 (1.05-19.36)		2.46 (0.78-7.81)		9.82 (2.27-42.51)		2.6 (1.16-5.79)	
**Combined LMW-E & CDK5 H-score**								
Both negative	Referent	0.0257	Referent	0.2854	Referent	0.0033	Referent	0.4086
Both positive	2.89 (1.14-7.34)		1.31 (0.8-2.15)		3.65 (1.54-8.66)		1.3 (0.7-2.39)	

* Combined I and II
Cox model fitting did not work for small sample size for tumor grade in Aci subtype

Supplementary Table 5. Univariable analysis for OS using variables LMW-E/CDK5 expression along with standard markers of clinical outcome. Tabular representation of univariable analysis of the indicated variables with overall survival in each subtype. Variables with p-values <0.05 indicate predictors of overall survival for each subtype, calculated using the Cox proportional-hazards model.

Overall survival	Aci		ADCC		MEC		SDC	
**Variable**	**n**	**Median Survival**	**n**	**Median Survival**	**n**	**Median Survival**	**n**	**Median Survival**
**No biomarkers**	73	137	202	124	82	241	101	26
**LMW-E H-score**								
0	53	273	106	136	60	371	38	35
1	16	48	64	117	20	43	54	26
**CDK5 H-score**								
0	47	273	89	181	47	375	40	29
1	22	77	83	107	32	90	52	21
**Combined LMW-E & CDK5 H-score**								
Both negative	43	273	69	187	43	375	28	36
Both positive	12	48	44	101	15	43	42	27
**Median survival is in months**								

Supplementary Table 6. Median OS as function of no biomarker or differential (+ or -) LMW-E and CDK5 expression. Summary of median OS focused on variables of no biomarker, LMW-E, CDK5 or LMW-E/CDK5 low (H-score =0) and high expression (H-score =1).

Variable	Aci- RFS Multivariable Analysis HR (95% CI)	Aci- RFS P-value	AdCC- RFS Multivariable Analysis HR (95% CI)	AdCC- RFS P-value	MEC- RFS Multivariable Analysis HR (95% CI)	MEC- RFS P-value	SDC- RFS Multivariable Analysis HR (95% CI)	SDC- RFS P-value
**Age (years)**	1 (0.96-1.03)	0.7619	0.97 (0.95-0.99)	**0.0032***	1.01 (0.98-1.04)	0.3749	1.03 (1-1.06)	0.055
**Sex**								
Female	Referent		Referent		Referent		Referent	
Male	0.59 (0.21-1.62)	0.3034	1.51 (0.97-2.35)	0.071	0.62 (0.23-1.64)	0.3344	0.97 (0.46-2.06)	0.9335
**Perineural invasion**								
No	Referent		Referent		Referent		Referent	
Yes	2.18 (0.84-5.71)	0.1109	0.97 (0.56-1.68)	0.907	0.98 (0.37-2.58)	0.9675	0.69 (0.32-1.51)	0.3597
**Lymphovascular invasion**								
No	Referent		Referent		Referent		Referent	
Yes	1.3 (0.39-4.36)	0.6737	2.4 (1.33-4.33)	**0.0035***	0.83 (0.23-3.05)	0.7806	1.81 (0.9-3.63)	0.0957
**Margin**								
Negative	Referent		Referent		Referent		Referent	
Positive	2.03 (0.8-5.19)	0.1372	1.1 (0.7-1.72)	0.6785	1.44 (0.41-5.01)	0.5686	1.79 (0.72-4.46)	0.212
**LMW-E H-score**								
0	Referent		Referent		Referent		Referent	
1	1.04 (0.38-2.84)	0.9374	1.78 (1.09-2.92)	**0.0217***	5.68 (1.74-18.49)	**0.0039***	1.57 (0.6-4.13)	0.3567
**CDK5 H-score**								
0	Referent		Referent		Referent		Referent	
1	1.93 (0.79-4.69)	0.1486	1.78 (1.07-2.98)	**0.0268***	0.7 (0.25-2)	0.5059	1.12 (0.49-2.55)	0.7861
**Clinical TNM stage**								
I/II	Referent		Referent		Referent		Referent	
III	2.87 (0.45-18.16)	0.2637	0.37 (0.07-1.83)	0.2214	2.42 (0.45-13.06)	0.3057	1.7 (0.23-12.86)	0.6064
IV	6.67 (1.59-27.98)	**0.0095***	3.07 (1.38-6.86)	**0.0061***	6.39 (2.04-19.97)	**0.0014***	10.36 (2.26-47.46)	**0.0026***
* indicates significance with p-value <0.05								

Supplementary Tables 7: Multivariable analysis for RFS using variables LMW-E/CDK5 expression along with standard markers of clinical outcome. Multivariable analysis for RFS by Cox proportional hazards modeling for each subtype. Variables with p-values <0.05 indicate independent predictors of recurrence- free survival for each subtype

Variable	Aci- OS		AdCC- OS		MEC- OS		SDC- OS	
	Multivariable Analysis		Multivariable Analysis		Multivariable Analysis		Multivariable Analysis	
	HR (95% CI)	P-value	HR (95% CI)	P-value	HR (95% CI)	P-value	HR (95% CI)	P-value
**Age (years)**	1.04 (1-1.09)	0.0795	1 (0.98-1.01)	0.6192	1.04 (1.01-1.08)	**0.0233***	1 (0.98-1.03)	0.7591
**Sex**								
Female	Referent		Referent		Referent		Referent	
Male	1.01 (0.36-2.8)	0.9894	0.86 (0.57-1.32)	0.5008	1.54 (0.68-3.49)	0.3026	0.63 (0.31-1.26)	0.1885
**Perineural invasion**								
No	Referent		Referent		Referent		Referent	
Yes	2.47 (0.87-7.07)	0.0905	1.25 (0.74-2.1)	0.4043	0.59 (0.21-1.69)	0.3265	1.1 (0.56-2.17)	0.7826
**Lymphovascular invasion**								
No	Referent		Referent		Referent		Referent	
Yes	3.22 (0.85-12.12)	0.0842	1.77 (0.99-3.16)	0.0543	0.66 (0.21-2.02)	0.4624	1.65 (0.85-3.21)	0.1428
**Margin**								
Negative	Referent		Referent		Referent		Referent	
Positive	0.88 (0.31-2.45)	0.8048	0.92 (0.59-1.42)	0.6988	1.5 (0.48-4.71)	0.4862	1.73 (0.73-4.14)	0.2163
**LMW-E H-score**								
0	Referent		Referent		Referent		Referent	
1	3.44 (1.03-11.48)	**0.0448***	0.93 (0.59-1.46)	0.7445	1.03 (0.35-3.03)	0.9604	0.82 (0.3-2.27)	0.7090
**CDK5 H-score**								
0	Referent		Referent		Referent		Referent	
1	0.41 (0.13-1.28)	0.1258	1.02 (0.64-1.61)	0.9365	0.98 (0.36-2.71)	0.9747	1.02 (0.45-2.32)	0.9625
**Clinical TNM stage**								
I/II	Referent		Referent		Referent		Referent	
III	5.54 (0.86-35.76)	0.0721	0.78 (0.3-2.04)	0.6067	1.98 (0.39-10.14)	0.4142	1.4 (0.34-5.76)	0.6385
IV	5.1 (0.93-27.85)	0.0601	1.7 (0.92-3.12)	0.0877	10.66 (3.42-33.22)	**<0.0001***	4.34 (1.3-14.55)"	**0.0172***
* indicates significance with p-value <0.05								

Supplementary Table 8. Multivariable analysis for OS using variables LMW-E/CDK5 expression along with standard markers of clinical outcome. Multivariable analysis for OS by Cox proportional hazards modeling for each subtype. Variables with p-values <0.05 indicate independent predictors of recurrence- free survival for each subtype.

Mouse model	Subtype of Salivary gland cancer	Time to tumor development	Clinical site of salivary tumors	Recapitulation of clinical features
Ela-CreERT-LGL-KRASG12D	SDC	15-20 days from induction with Doxycycline	Parotid gland (100% frequency)	Histologically similar to SDC. However, pancreatic fibrosis (which precedes PDAC*) was seen in 100% of the mice
MMTV APC-/- Pten-/-	Aci	2-6 months post induction with Doxycycline	Parotid gland (23% frequency)	Histologically similar to Aci. However, WNT signaling is needed for tumor development; clinically which has shown to have no role in Aci
MMTV-RANKL	Poorly differentiated adenocarcinomas	1 year post induction with Doxycycline	Parotid gland (Frequency unclear)	RANKL and RANK have no significant correlation to clinical parameters. There is very little to no evidence of this model recapitulating clinically relevant features of salivary gland cancers
MMTV-LMW-E p53+/- CDK2-/- (current study)	Intermediate to high grade adenocarcinomas	6-12 months post induction with Doxycycline	Parotid gland (25% frequency)	Histologically recapitulates intermediate to high grade tumors of the parotid gland and predictive of recurrence free survival.

Supplementary Table 9. Comparison of different salivary gland cancer mouse models. Summary of different mouse models in the field compared to the current study (grey highlighted row). References have been added to the main text (discussion section) for each model.
* PDAC= pancreatic ducal adenocarcioma
