# Supplementary material for: LMW cyclin E and its novel catalytic partner CDK5 are therapeutic targets and prognostic biomarkers in salivary gland cancers
Source: Oncogenesis. 2021 May 14;10(5):40. doi: 10.1038/s41389-021-00324-z (PMC8121779; doi:10.1038/s41389-021-00324-z)
Supplement: Supplementary file 3 — Supplementary Figures and Figures legends [file 41389_2021_324_MOESM3_ESM.pdf]

A.

CDK2 status	ID	Histologic pattern	Mitoses #/10 hpf	Cytomegaly/ karyomegaly (cells/10 hpf)	Necrosis (%)
+/+	3082	Trabecular, Acinar	47	1	10%
+/+	3085	Trabecular, Acinar ,Solid	100+	0	20%
+/+	3088	Trabecular, Acinar,Solid	2	31	10%
+/-	5574	Acinar	100+	0	20%
+/-	4032	Trabecular, Acinar, Solid	100+	0	30%
+/-	4055	Trabecular, Acinar, Solid	100+	32	5%
+/-	4107	Trabecular, Acinar	100+	1	5%
+/-	4757	Trabecular, Acinar	100+	2	30%
-/-	4034	Solid, Acinar	7	0	0
-/-	4457	Solid, Trabecular	50	0	30%
-/-	5188	Acinar, Solid	100+	0	10%

B.

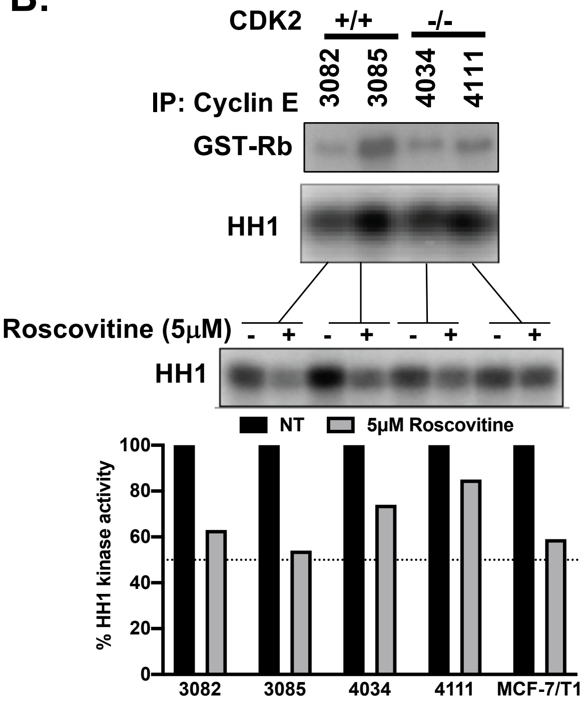

C.

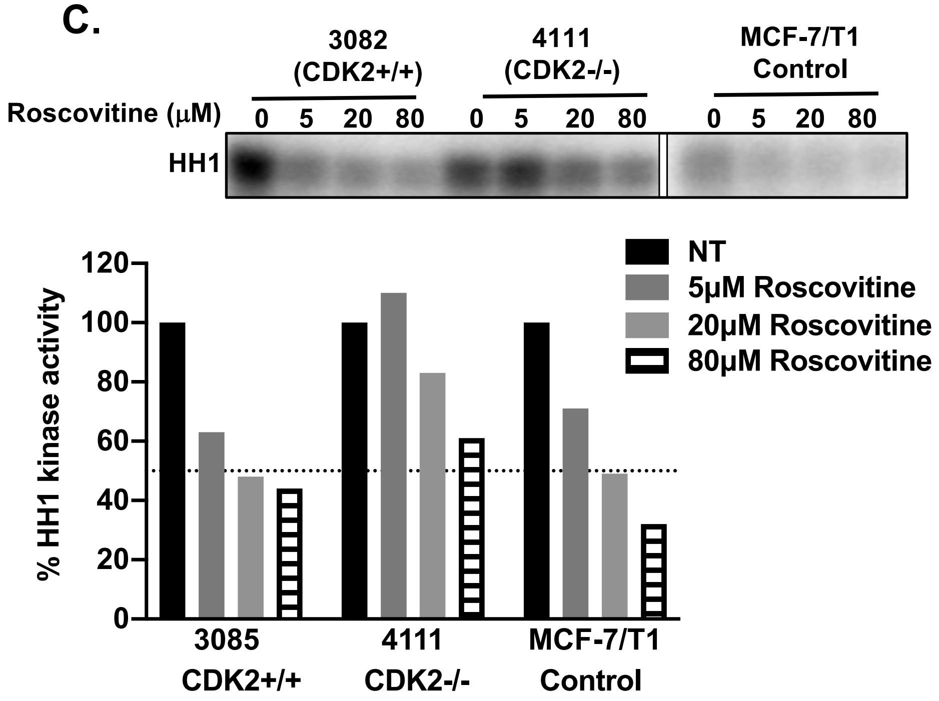

Supplementary Figure 1.

Supplementary Figure 1 Salivary gland tumors from CDK2 knockout animals retain CDK1 kinase activity. A; Summary of histological patterns, mitotic indices and other histological features of salivary gland tumors obtained from MMTV-LMW-E-T1-p53+/- mice with varying CDK2 status (CDK2+/+, CDK2+/-, and CDK2-/-). **B.** (*Upper gels*) Cyclin E associated kinase assays HH1 and GST-Rb substrates following IP with cyclin E, using 250 µg protein extracts of the indicated CDK2+/+ and CDK2-/- tumors. (*Lower gel*) Kinase assays with HH1 substrate following treatment with 5µM roscovitine and IP with cyclin E, using 250 µg protein extracts of the indicated CDK2+/+ and CDK2-/- tumors. Percent kinase activity is normalized to untreated samples and represents the mean of 2 independent experiments. **C.** Kinase assays with HH1 substrate following treatment with 5-80µM roscovitine and IP with cyclin E, using 250 µg protein extracts of the indicated CDK2+/+ and CDK2-/- tumors. Percent kinase activity is normalized to untreated samples and represents the mean of 2 independent experiments.

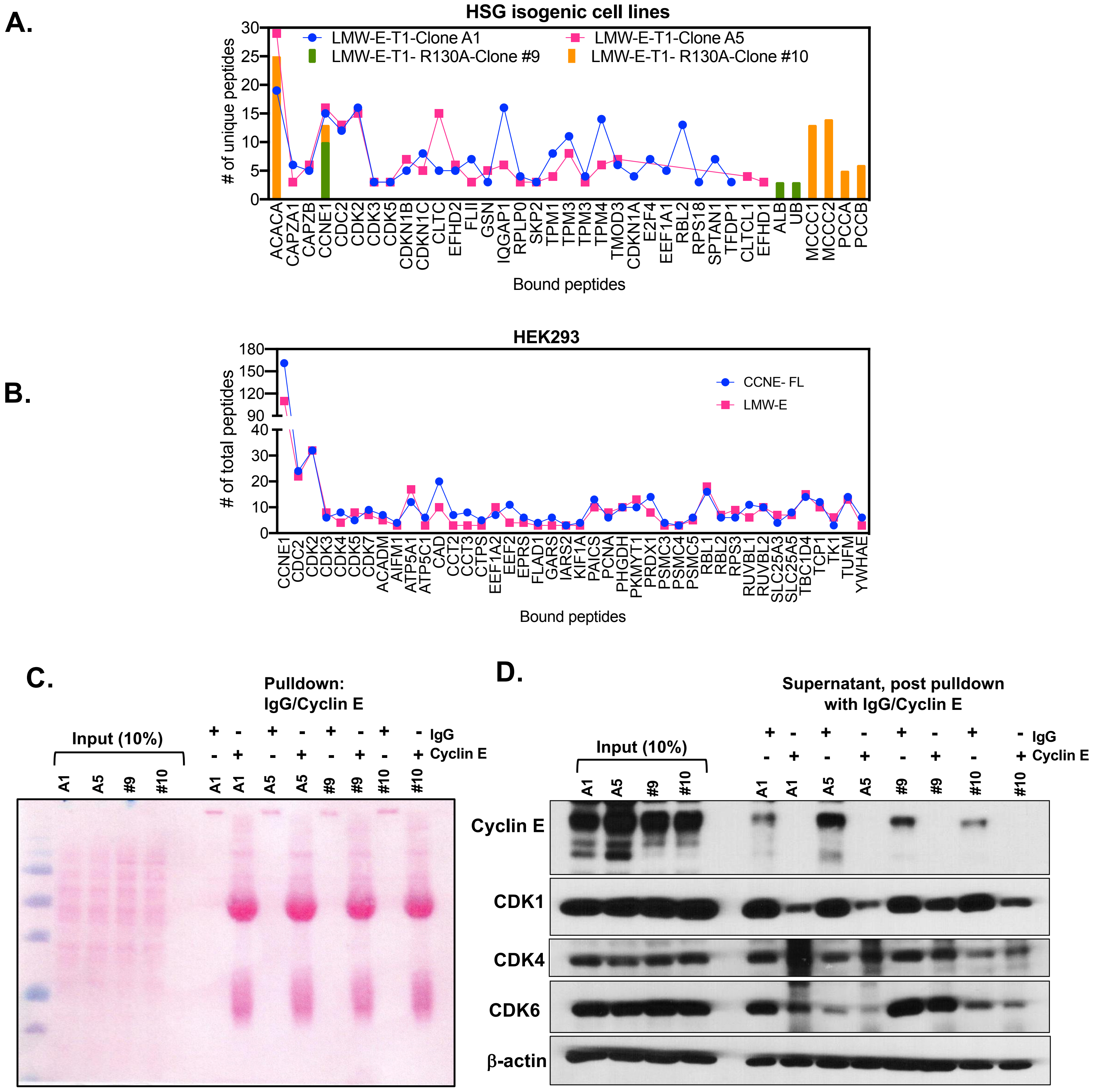

Supplementary Figure 2.

Supplementary Figure 2: Mass Spectrometry and IP/western blot analysis reveals CDK5 as a binding partner for cyclin E A. Isogenic HSG cell lines were subject to immunoprecipitation (IP) with an anti-flag antibody using 1 mg of protein extracts followed by flag peptide elution and loading (20% of eluent) onto a 10% SDS-PAGE followed by mass spectrometry analysis. The graph shows the number of unique peptides of each protein bound to two LMW-Cyclin E expressing clones A1 and A5 (blue and pink lines respectively). Similarly, bar graphs show peptides unique to LMW-E^{R130A} clones 9 and 10 (green and orange bars respectively). **B.** Mass spectrometry results indicating the number of unique peptides of different proteins bound to either cyclin E-FL or LWM-E expressing HEK293 cells. **C.** Ponceau stained membranes of IP/Western blots shown in main Figure 2E. Equal amounts of IgG bands seen in the cyclin E pulldown lanes indicate equal loading of samples. **D.** Western blot analysis of supernatants to asses for unbound proteins, obtained from the IP/Western blots shown in main Figure 2E. Detection of signal in the cyclin E pulldown lanes indicate unbound proteins and thus non-binding to cyclin E. Lanes labelled IgG lanes indicate pulldown with anti-IgG and lanes labelled LMW-E indicate pulldown with anti-cyclin E

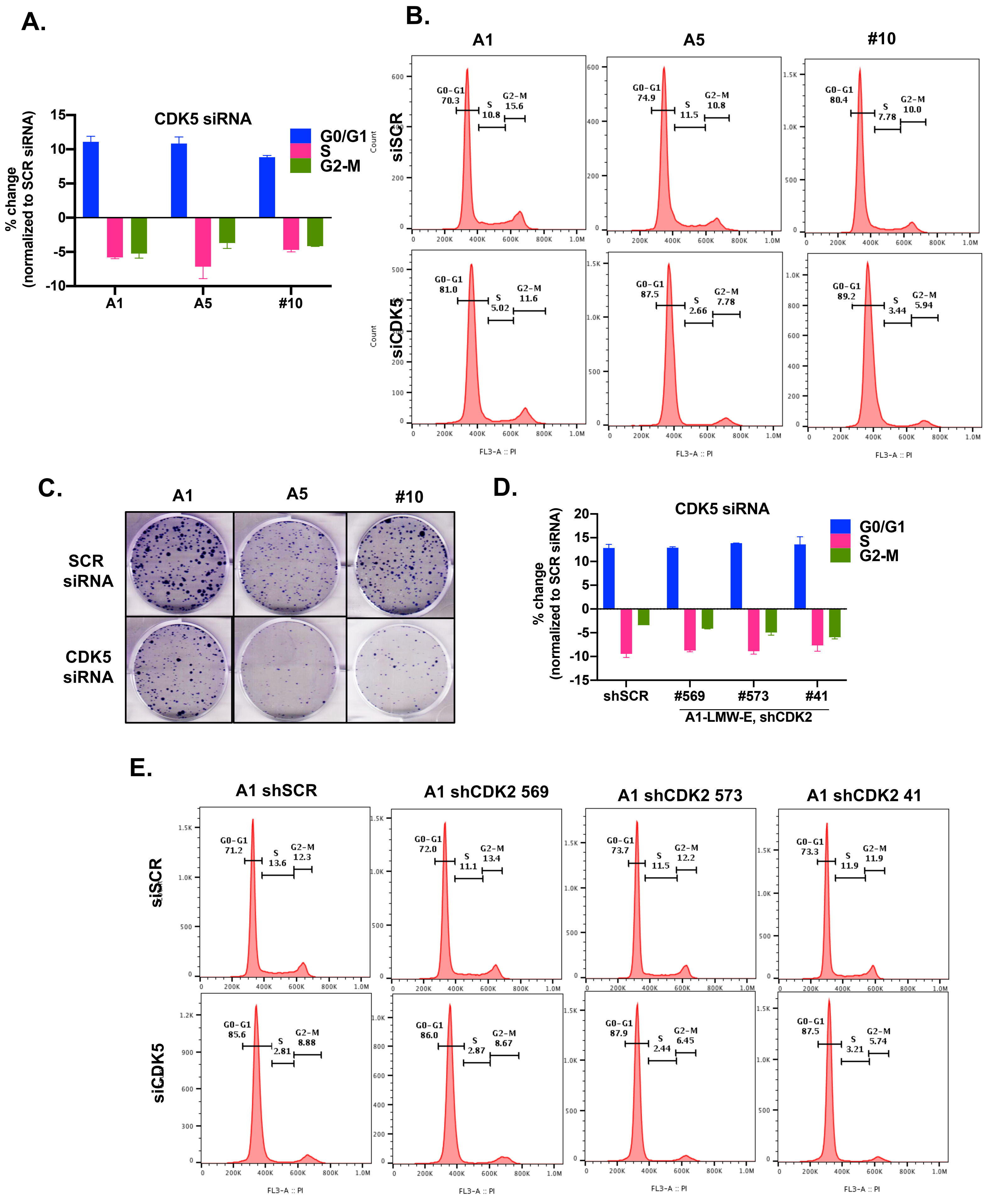

Supplementary Figure 3.

Supplementary Figure 3. Cell cycle analysis in cells with transient knockdown of CDK5

Cell cycle profiles, representative histograms, and representative colony formation images of isogenic HSG cell lines (A1, A5, clones 9 and 10) **(A, B, C)** was assessed post CDK5 knockdown, done by transiently transfecting with either 50nM of SCR siRNA or CDK5 siRNA in each cell line indicated. 72-hours post transfection, cells were harvested, fixed, stained with PI and analyzed by flow cytometry for **A and B**. Colony formation images in **C** are representative of 12 days post SCR/CDK5 siRNA transfection **(D, E)** Cell cycle profiles and representative histograms of A1 isogenic cell lines (-/+ shCDK2 knockdown), under identical experimental conditions as described for figures A and B.

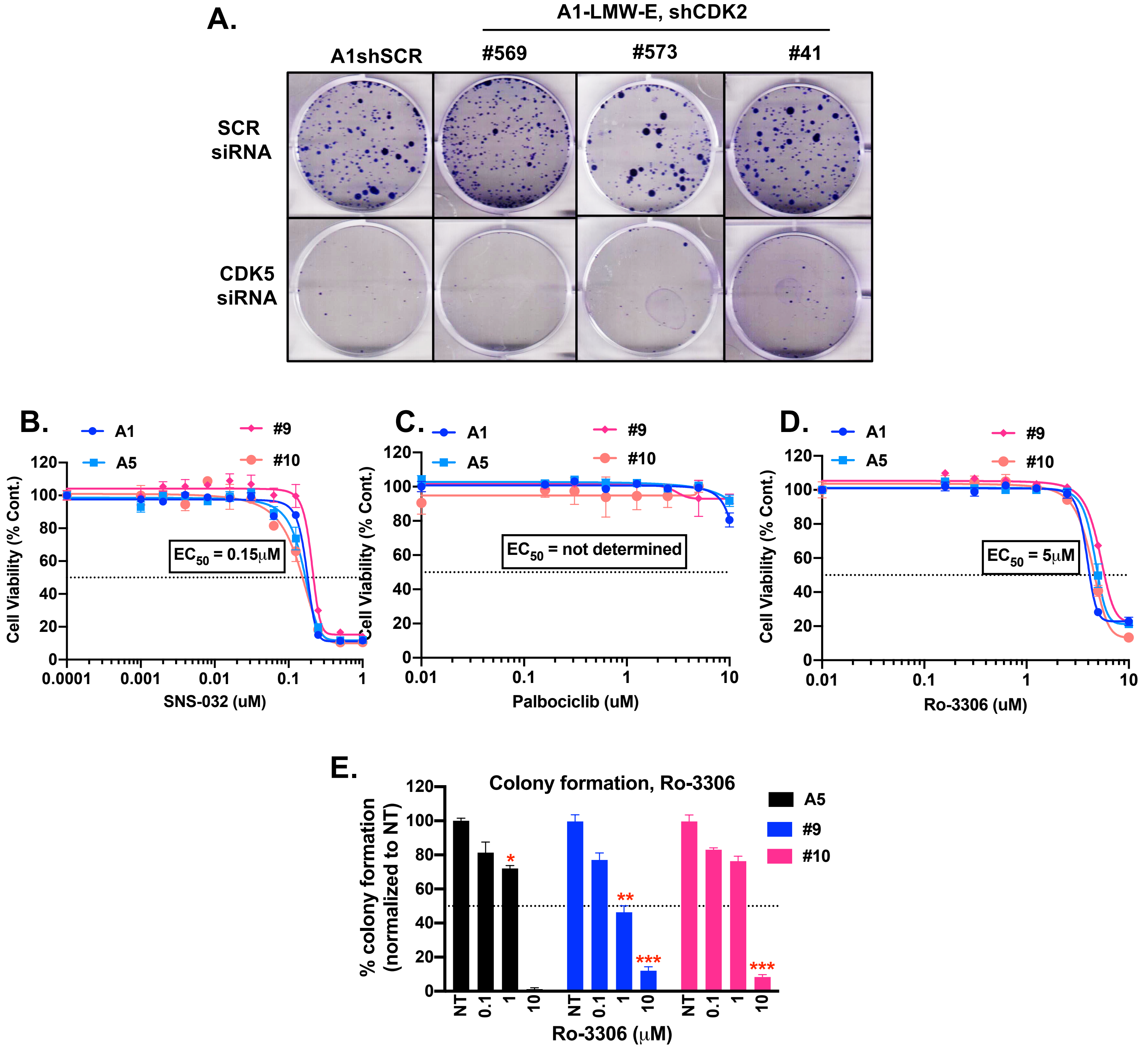

Supplementary Figure 4.

Supplementary Figure 4: CDK5 is the main LMW-E bound kinase, required in LMW-E SGT cell lines: **A**. Representative colony formation images of A1 isogenic cell lines (-/+ shCDK2 knockdown) post CDK5 knockdown, done by transiently transfecting with either 50nM of SCR siRNA or CDK5 siRNA in each cell line indicated. Images in **A.** are representative of 12 days post SCR/CDK5 siRNA transfection **B, C, D.** Isogenic HSG cell lines (LMW-E expressing A1 and A5, LMW-E-R130A expressing clones 9 and 10) were plated in 96-well plates at a density of 100 cells/well. Cells were treated with **B.** 0-1μM CDK2 inhibitor SNS-032, **C.** 0-10μM CDK4/6 inhibitor Palbociclib and **D.** 0-10μM CDK1 inhibitor Ro-3306 (triplicates/sample/dose) for 12 days. Media was replenished every 3 days. Effects on cell proliferation was assessed using CellTiterBlue and presented as dose response curves. **E.** Isogenic HSG cell lines (LMW-E expressing A1 and A5, LMW-E-R130A expressing clones 9 and 10) were treated with 0.1μM, 1μM and 10μM (triplicates/sample/dose) of Ro-3306 for 3 days. Thereafter, cells were collected and plated in 6- (colony forming assays) well plates at a density of 500 cells/well for 12 days with drug. Media was replenished every 3 days. Effect on cell proliferation was assessed using crystal violet staining, colonies were enumerated and presented as bar graphs respectively. % colony proliferation represents the mean of 3 independent experiments. p-values (* p <0.05, ** p<0.01, *** p<0.001) were calculated using the unpaired t-test with Welch's correction.

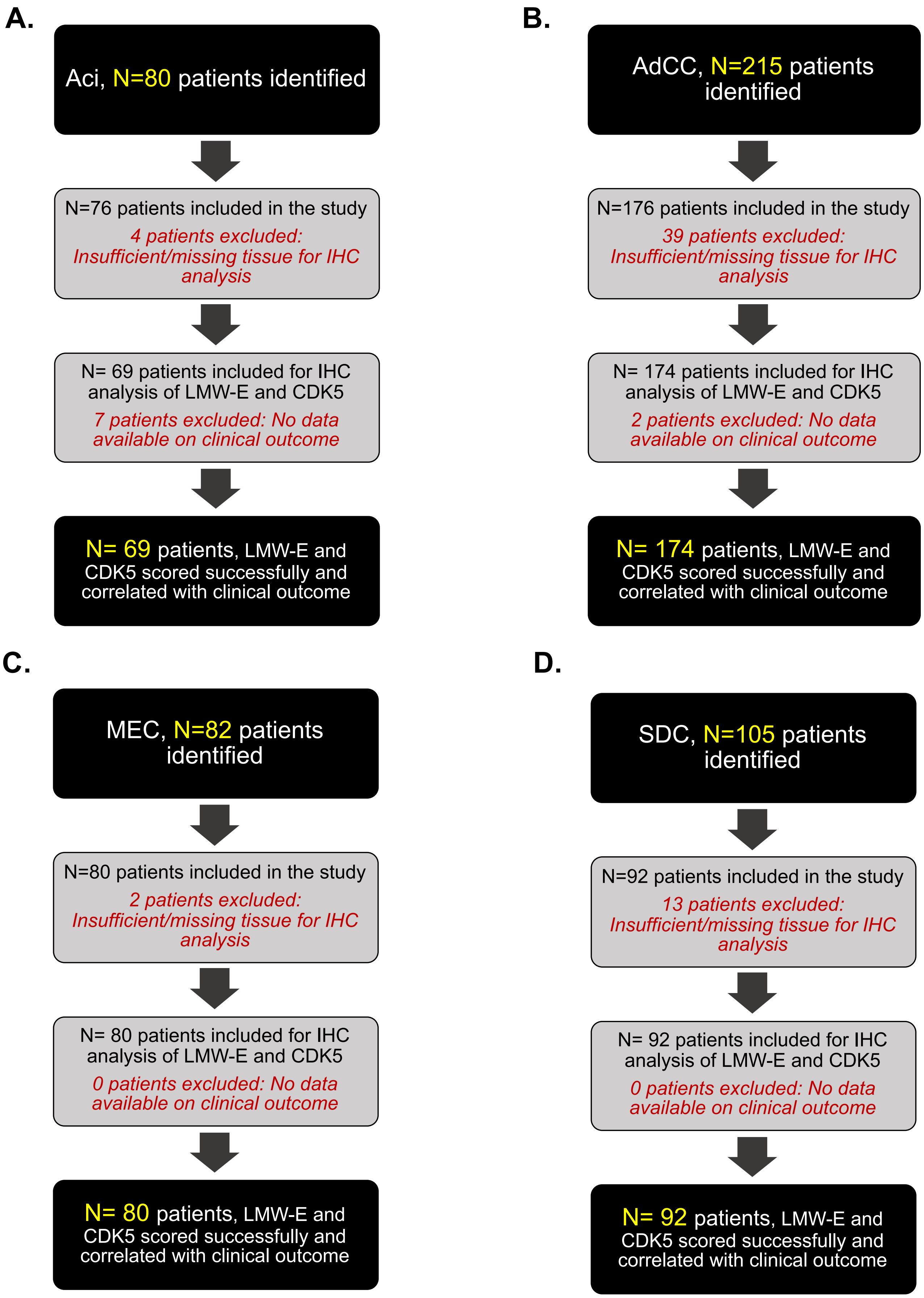

Supplementary Figure 5.

Supplementary Figure 5. Flowcharts of patients included in the study: A total of 482 patients were assessed for eligibility, divided under the subtypes of Aci (N=80) **(Panel A)**, AdCC (N=215) **(Panel B)**, MEC (N=82) **(Panel C)** and SDC (N=105) **(Panel D)** respectively. A total of 67 patients were excluded due to insufficient or missing tissue for IHC analysis or lack of availability of outcome data due to loss of follow up. This led to 415 patients being eligible for the study, divided under the subtypes Aci (N=69) **(Panel A)**, AdCC (N=174) **(Panel B)**, MEC (N=80) **(Panel C)** and SDC (N=92) **(Panel D)** respectively.

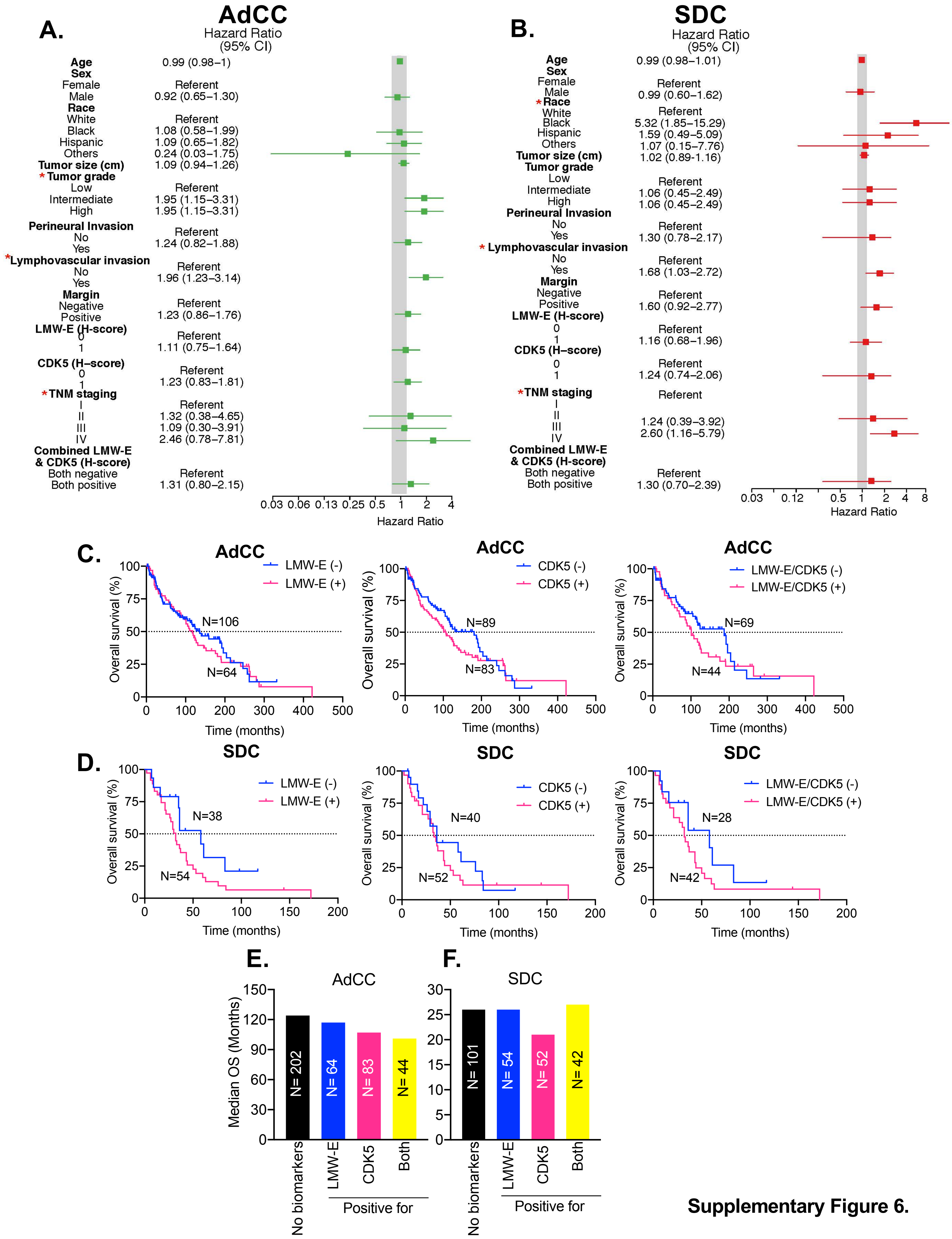

Supplementary Figure 6.

Supplementary Figure 6. LMW-E and CDK5 are not associated with overall survival in AdCC and SDC subtypes of salivary gland tumors. **A, B.** Forest plots indicating univariable analysis of the indicated variables with overall survival in each subtypes AdCC and SDC. The solid grey line indicates a hazard ratio of 1 for the overall population. Variables with p-values <0.05 are marked with * and are predictors of overall survival for each subtype, calculated using the Cox proportional-hazards model. **C, D.** Kaplan-Meier plots for subtypes AdCC and SDC respectively, according to LMW-E, CDK5 or LMW-E/CDK5 expression. The combination of LMW-E and CDK5 was showed no significant association with overall survival in these. **E, F.** Graph comparing median OS without any biomarkers to that when patient tumor samples are positive (H-score =1) for LM LMW-E, CDK5 or LMW-E /CDK5; for every subtypes AdCC and SDC.
